# Applications of Bayesian network models in predicting types of hematological malignancies

**DOI:** 10.1038/s41598-018-24758-5

**Published:** 2018-05-03

**Authors:** Rupesh Agrahari, Amir Foroushani, T. Roderick Docking, Linda Chang, Gerben Duns, Monika Hudoba, Aly Karsan, Habil Zare

**Affiliations:** 1 Department of Computer Science, Texas State University, San Marcos, Texas 78666 USA; 2 Michael Smith Genome Sciences Centre, British Columbia Cancer Agency, Vancouver, British Columbia V5Z 1L3 Canada; 3 Department of Pathology and Laboratory Medicine, Vancouver General Hospital, Vancouver, British Columbia V5Z 1M9 Canada; 4 Department of Cell Systems & Anatomy, The University of Texas Health Science Center, San Antonio, Texas 78229 USA

## Abstract

Network analysis is the preferred approach for the detection of subtle but coordinated changes in expression of an interacting and related set of genes. We introduce a novel method based on the analyses of coexpression networks and Bayesian networks, and we use this new method to classify two types of hematological malignancies; namely, acute myeloid leukemia (AML) and myelodysplastic syndrome (MDS). Our classifier has an accuracy of 93%, a precision of 98%, and a recall of 90% on the training dataset (*n* = 366); which outperforms the results reported by other scholars on the same dataset. Although our training dataset consists of microarray data, our model has a remarkable performance on the RNA-Seq test dataset (*n* = 74, accuracy = 89%, precision = 88%, recall = 98%), which confirms that eigengenes are robust with respect to expression profiling technology. These signatures are useful in classification and correctly predicting the diagnosis. They might also provide valuable information about the underlying biology of diseases. Our network analysis approach is generalizable and can be useful for classifying other diseases based on gene expression profiles. Our previously published *Pigengene* package is publicly available through Bioconductor, which can be used to conveniently fit a Bayesian network to gene expression data.

## Introduction

Acute Myeloid Leukemia (AML) is a cancer of the myeloid blood cells in which bone marrow produces abnormal white blood cells, abnormal red blood cells, or abnormal platelets. It primarily affects the elderly, and it is the most common acute leukemia among adults. It is an aggressive type of blood cancer, which accounts for about 1.2% of the total cancer deaths in the U.S.^1^.

Myelodysplastic Syndrome (MDS) is a disease that affects myeloid cells in the bone marrow and the blood. MDS is characterized by abnormal hematopoiesis, which is the ineffective production of blood cells and platelets in the bone marrow^2^. In contrast to AML, MDS is relatively mild and has a low mortality risk, but it can progress over time and 30% of all MDS cases will ultimately develop into AML^3,4^. Therefore, it is important to compare these two diseases and provide biological insights into their similarities and differences at the molecular level.

**Figure 1:**
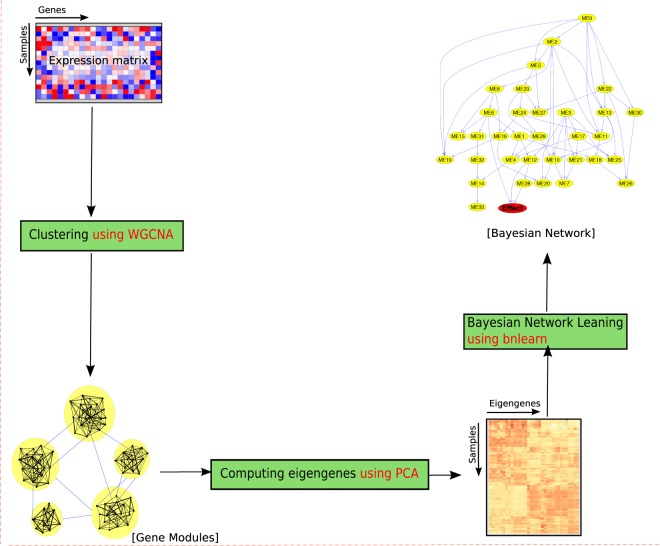
Schematic view of the methodology. **(A)** The input is the gene expression profile (matrix). **(B)** We applied *WGCNA* to build the coexpression network and to identify gene modules (clusters). **(C)** PCA is used to summarize the biological information of each gene module into an eigengene. **(D)** A BN is fitted to the eigengenes to delineate the relationships between modules. We also used the fitted BN as a probabilistic predictive model. The tools used for each step are highlighted in red.

Accordingly, we compared the gene expression profiles of AML and MDS using network analysis. The goal of this study was to improve the classification of these two hematological malignancies solely based on gene expression data. This study is inspired by, and builds upon, the coexpression network analysis and Bayesian network (BN) model. Figure 1 shows the schematic overview of our methodology.

We used $\underline{{\rm{w}}}{\rm{eighted}}\,\underline{{\rm{g}}}{\rm{ene}}\,\underline{{\rm{c}}}{\rm{oexpression}}\,\underline{{\rm{n}}}{\rm{etwork}}\,\underline{{\rm{a}}}{\rm{nalysis}}$ weighted gene coexpression network analysis (*WGCNA*)^5^ to group related genes into gene modules (clusters) based on their coexpression patterns in AML. *WGCNA* uses the average linkage hierarchical algorithm to cluster the genes^6^. For each gene module, *WGCNA* computes one eigengene, which summarizes the biological information in that module into one value per sample^7^. We used these eigengenes to train a Bayesian network (BN) in which nodes (random variables) represent gene modules, and the directed edges (arcs) represent the conditional dependencies between the eigengenes.

Bayesian networks have been used to model gene expression data^8–15^ and gene regulatory networks^16–20^. A BN consists of a directed acyclic graph (DAG)^21,22^ and a set of corresponding conditional probability density functions. The structure of a DAG is defined by two sets: the set of nodes (vertices), which represent random variables, and the set of directed edges. In a DAG, if a directed edge extends from node X to another node Y, then X is designated a *parent node* of Y, and Y becomes a *child node* of X. The directed edges in a BN structure model the dependencies between the variables (nodes)^23^. In particular, the joint probability density function of the random variables in a BN can be written as a product of the individual density functions, conditional on their parent variables^24^.

Different variations of the BN model have been used to analyze gene expression data^8^, including the naïve Bayes classifier (NB)^25,26^, the Bayesian network augmented naïve Bayesian classifier (BAN)^27^, the k-dependence Bayesian classifier (KDB)^28^, and the general Bayesian network model^29^. In this study, we use a general Bayesian network in which each node is an observed random variable that models the expression value of an eigengene.

## Results

**Figure 2:**
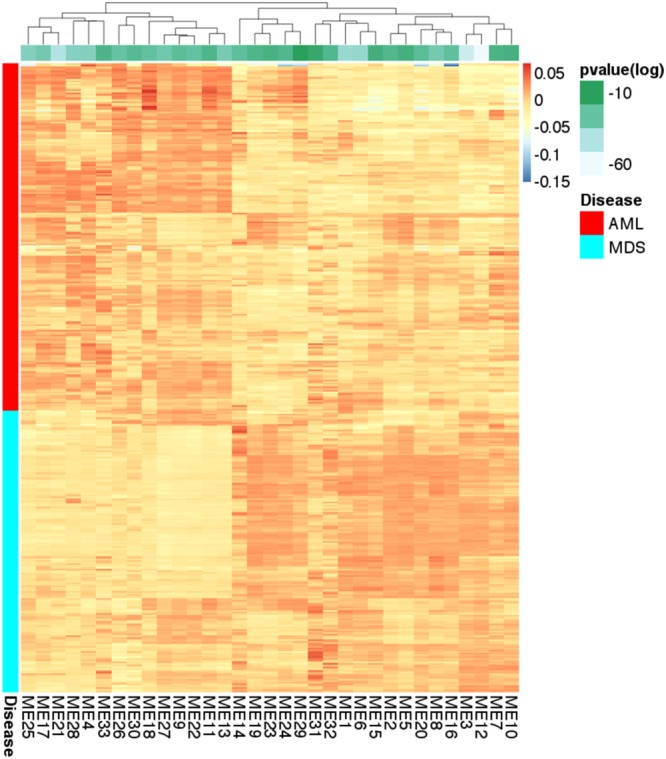
Expression of eigengenes in the MILE dataset. Each row corresponds to a sample. Modules (columns) are clustered based on the similarity of expression in the MILE dataset. The majority of eigengenes show a different pattern of expression in the two diseases. The green strip at the top shows the adjusted p-values of Welch’s t-tests in logarithmic scale (base 10). The adjusted p–values are in the order of 10^−60^ to 10^−10^, which indicates that the eigengenes are highly discriminative features.

**Figure 3:**
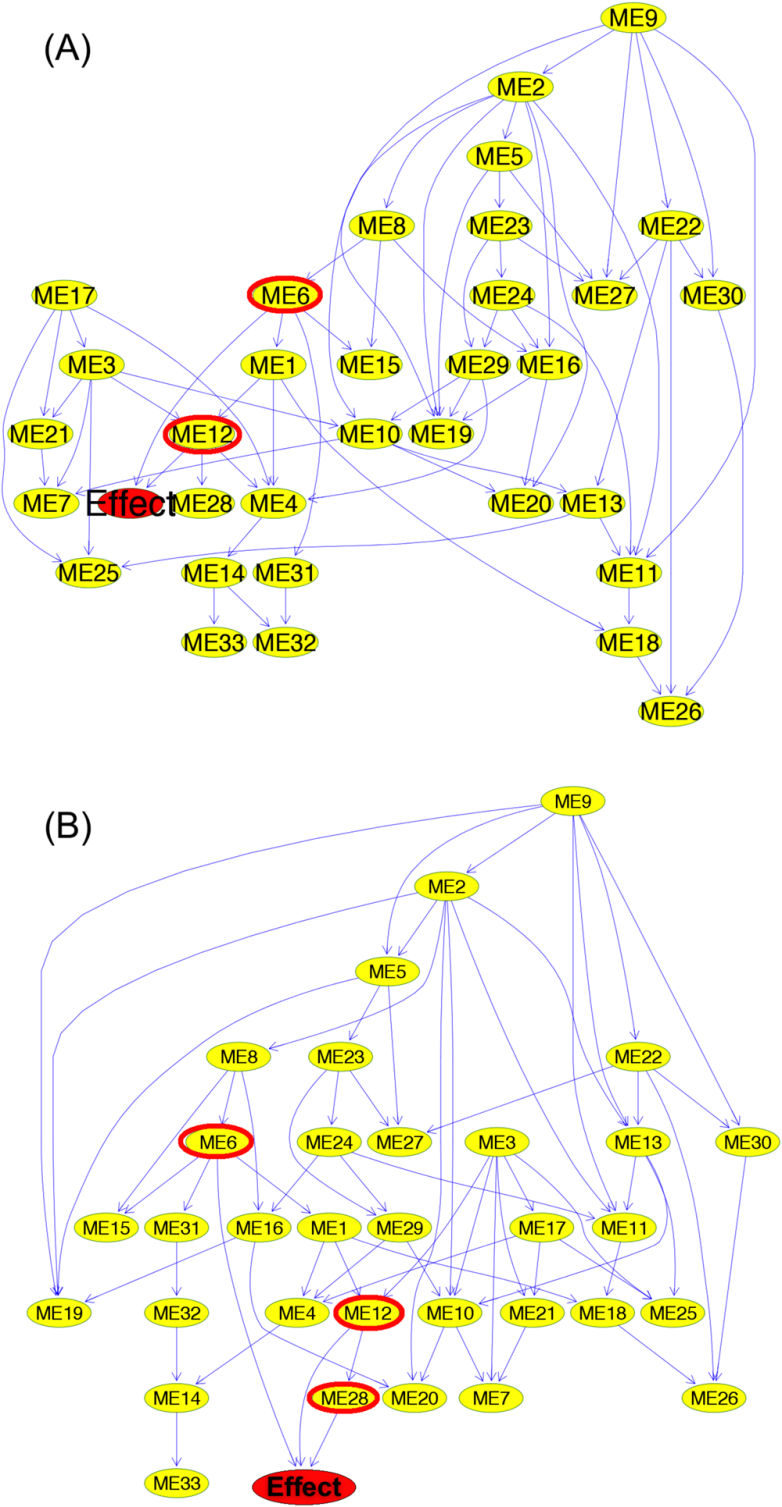
Consensus BN structures. Each yellow node represents an eigengene. The *Effect* node is a binary variable that models the disease type. Its parents are denoted by red circles. The directed edges (arcs) model the probabilistic dependencies between nodes. Although these consensus networks are obtained from 500 **(A)** and 5, 000 **(B)** BNs, they have fairly similar structures.

The majority of the 33 inferred eigengenes are differentially expressed in AML versus MDS in the MILE dataset (Fig. 2). We hypothesized that these eigengenes are important biological signatures that can predict disease types solely based on gene expression. To validate this hypothesis, we modeled the probabilistic dependencies between the eigengenes using a BN (Fig. 3). We used Bayesian networks as probabilistic predictive models to determine the type of the disease.

### The performance of the predictive model on the MILE (training) dataset

We considered AML cases as positive samples and considered MDS cases as negative samples, and computed the performance of the predictions in both training (MILE) and test (BCCA) datasets. Supplementary File S4 shows the confusion matrices and performance of all 5 models obtained from our subsampling. On the MILE dataset, the accuracy of predictions on the training partitions is in the range of 88%–97%, with an average accuracy of 93.2%. The accuracy on the validation partition is in the range of 78.5%–94.6%, with an average accuracy of 88% (Table 1). The average performance on the training partitions (i.e., accuracy = 93%, precision = 90%, recall = 95%) is comparable to the performance of majority vote of the models (i.e., accuracy = 93%, precision = 98%, recall = 90%). Both of these predictions are better than the predictions achieved by Mills *et al*.^30^. on the same MILE dataset (i.e., accuracy = 74%, precision = 70%, recall = 93%), which were predictions based on margin trees^31,32^.

**Table 1:** Performance of predictions made by individual models on the training and validation partitions of the MILE dataset, which has 202 positive (AML) and 164 negative (MDS) samples.

Model	Training partition			Validation partition		
	Accuracy (%)	Precision (%)	Recall (%)	Accuracy (%)	Precision (%)	Recall (%)
Model 1	96.9	95.7	98.7	78.4	71.8	84.8
Model 2	92.8	89.6	97.3	89.2	84.2	94.1
Model 3	91.5	88.8	87.4	86.5	80.1	94.5
Model 4	88.0	83.1	94.3	94.6	95.2	95.2
Model 5	96.6	96.9	96.9	91.5	95.1	90.1
**Mean**	**93.2**	**90.8**	**94.9**	**88.0**	**85.2**	**91.8**

Each model was trained using a subsample from the MILE dataset, which consists of four fifth of training cases (Supplementary File S2).

### The performance of the predictive model on the BCCA (test) dataset

The MILE dataset, which was used to train the predictive model in this study, was assayed using microarrays. To rigidly evaluate our predictive model, we measured its performance on the BCCA dataset, which was assayed using RNA-Seq. The following results indicate that our predictive model is robust and accurate, although the profiling technologies that were used to measure the gene expressions in the MILE and BCCA datasets are different.

We inferred the 33 eigengenes in the BCCA dataset (Methods) and used them to infer the disease types of the 74 test samples (Table 2). We used the BN models that were trained on the MILE dataset *without changing any parameter*. Also, the BCCA dataset did not have any contribution in learning the BN structures and the coexpression network. Out of 52 AML and 22 MDS cases, majority voting misclassified 7 MDS samples as AML and only 1 AML sample as MDS, which results in an accuracy of 89%, a precision of 88% and a recall of 98% on the test dataset. This performance shows that our predictive model was not overfitted to MILE (the training) dataset.

**Table 2:** Performance of predictions on the BCCA dataset, which has 52 positive (AML) and 22 negative (MDS) samples.

Model	Accuracy (%)	Precision (%)	Recall (%)
Model 1	90.5	92.5	94.2
Model 2	71.6	84.4	73.1
Model 3	86.5	86.2	96.2
Model 4	87.8	85.3	100
Model 5	83.8	85.7	92.3
**Mean**	**84.0**	**86.8**	**91.2**
**Majority vote**	**89.2**	**87.9**	**98.1**

The majority vote performs better than the individual BN models. Each model was trained using a subsample from the MILE dataset, which consists of four fifth of training cases (Supplementary File S2).

### Comparison with the SVM

We compared the performance of our predictive model versus a support vector machine (SVM)^33–35^. From the common kernels (i.e., linear, polynomial, and Gaussian radial base functions), we chose Gaussian radial, which was shown by Brown *et al*.^36^ to be appropriate for analyzing gene expression data. First, instead of the 33 eigengenes, we used the top 33 differentially expressed genes (Supplementary Fig. S4) as features to fit an SVM to the MILE dataset (R package *e1071* Version 1.6–7^37,38^). While the resulting classifier showed a high accuracy of 98% on the training dataset, it performed poorly on the test, as it predicted all samples in the BCCA dataset to be AML. We obtained the same results on the test dataset when we increased the number of features to 600 genes, although the accuracy on the training dataset increased to 99%. This indicates that the SVM was already overfitted to the training data and including more genes would not be useful. Although using more features (genes) provides the model with more information, SVM cannot efficiently use this information because the degree of freedom (i.e., the number of parameters that must be learned from the data) also increases. This is a well-studied phenomenon, often referred to as “the curse of dimensionality” in the machine learning community^39–41^.

**Figure 4:**
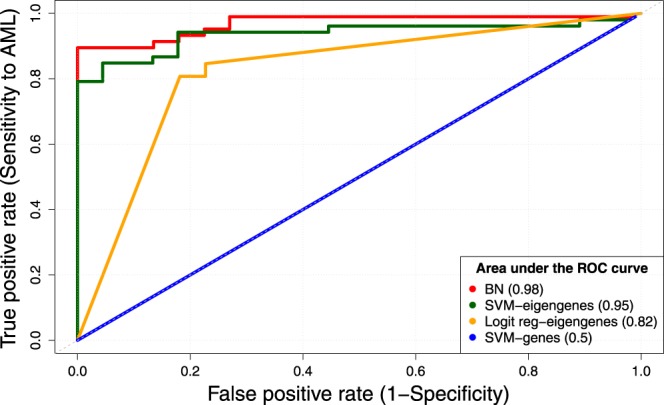
ROC curves. The predictions from the Bayesian network approach (red) leads to the highest AUC. The curve corresponding to the SVM predictions (green) is close to the best curve when eigengenes are used as features.

In contrast, by using the 33 eigengenes (i.e., the first principal components of every module) as features, we obtained an SVM with much higher performance on the test dataset; it reached an accuracy of 89%, a precision of 92%, a recall of 92% (Table 3), and an AUC of 0.95 (Fig. 4).

**Table 3:** Performance of SVM classifiers.

Classifier	Accuracy (%)	Precision (%)	Recall (%)
Radial using 33 genes	67.7	67.7	100
Radial using 600 genes	67.7	67.7	100
Linear using eigengenes	77	84.6	83
Polynomial using eigengenes	75.7	94.2	76.6
**Radial using eigengenes**	**89.2**	**92.3**	**92.3**
**Majority vote of BNs**	**89.2**	**87.9**	**98.1**

These SVM classifiers were trained using the 336 samples in the MILE dataset and were tested using the 52 AML and 22 MDS samples from the BCCA dataset. Among all kernels used on eigengenes, the Gaussian radial has the best performance as expected^36^, which is comparable with the majority vote of BN models. We used the polynomial kernel with degree 3 (e1071’s default value).

The SVM model misclassified only 8 (11%) samples, and it demonstrated a similar accuracy to the majority vote of the five BNs. The dramatic improvement in the performance of the SVM classifier, from 68% when using single genes to 89% using eigengenes, shows that eigengenes are informative and robust biological signatures. Interestingly, the AUC of a simple logistic model based on eigengenes is better than that of an SVM that uses the expression of individual genes as features (0.82 vs. 0.5, Fig. 4). These results suggest that eigengenes are preferable to individual differentially expressed genes for the analysis and comparison of the MILE and BCCA gene expression profiles.

### Analyzing the BN structures

We obtained five BN models from our subsampling approach (Supplementary File S2). Because of the Markov property of the Bayesian networks (Methods), the parents of the *Effect* node are the modules most related to, and predictive of, the disease type. Nine modules were among the parents of the *Effect* node in at least one BN (Table 4). The most frequent were Modules 4 and 12, which contain 332 and 113 genes, respectively. These modules were the parents of the *Effect* node in four BNs; therefore, they should be enriched with the genes that are associated with AML or MDS. Future pathway and functional analyses can determine the specific role of these genes in myeloid malignancies and explain how they differentiate between the two diseases.

**Table 4:** Parents of the *Effect* node in the 5 BNs that were fitted to the MILE dataset (Supplementary File S2).

Module	**4**	**12**	**1**	**28**	**30**	**3**	**6**	**14**	**21**
Frequency	4	4	3	2	2	1	1	1	1

### Generalizability to studying other diseases

The described methodology could also be applied to analyze other cancers. To demonstrate this, we analyzed 1,173 ER+ cases from the METABRIC^42^ breast cancer datasets. Specifically, we used 640 ER+ cases from the METABRIC discovery dataset for training a BN. We evaluated the resulting model on 533 different cases from the METABRIC validation dataset (Methods). We considered the cases who died due to breast cancer as positive samples. On the training dataset, the accuracy of predictions is 78%, where the precision is 91% and the recall is 81% (Supplementary File S6). On the test dataset, the accuracy of predictions is 70%, where the precision is 78% and the recall is 78%. The performance of the BN analysis is slightly better compared to SVM (i.e., accuracy = 68%, precision = 80%, recall = 76%). While SMV predictions are more precise by 2%, the recall of the BN approach is 2% higher than SVM.

In all the five BNs that were fitted to a portion of the training data, a module with 408 genes is the only parent of the *Effect* node (Supplementary Table S2). This module has a significant overlap with the Interleukin (IL) 9 signaling pathway. The overlap includes the following five genes: *IRS1*, *IRS2*, *STAT1*, *STAT5A*, and *VCP* (adjusted p–value of the hypergeometric test <0.04^43^), which are among the 24 genes that are annotated to be part of the IL–9 pathway according to NetPath^44^. Other groups have reported that the IL–9 signaling pathway can have a role in breast cancer progression^45–47^. This module has also a significant overlap with the IL–2 signaling pathway. Specifically, eight genes (i.e., *CRKL*, *HSP90AA1*, *IRS1*, *IRS2*, *SGK1*, *SHB*, *STAT1*, and *STAT5A*) are among the 81 genes that are annotated to be part of the IL–2 pathway (adjusted p–value of the hypergeometric test <0.04). Other studies have shown that the activation of the IL–2 signaling pathway is associated with proliferation of breast cancer cells^48,49^. Zaman *et al*. performed an integrative network analysis on 11 breast cancer cell lines, and identified 2,003 genes as potential driver–mutating genes^50^. The module of 408 genes that we identified in the current study has insignificant overlap with these 2,003 cancer hallmarks (i.e., 39 genes are in common, p-value of the hypergeometric test >0.3).

## Discussion

### Significance of network analysis

Biological processes in a cell often require coordination between multiple genes. Network analysis can detect subtle but coordinated changes in a set of interacting and functionally related genes^51–53^. Therefore, network analysis has advantages over conventional approaches that are based on a list of differentially expressed genes^54,55^. In particular, a coexpression network models the interaction between a large number of genes based on their coexpression pattern^5,56^. The resulting eigengenes, the features that summarize the biological information of modules, are robust with respect to noise and the profiling platform^53,57^. This is evident from our experiment that compared the performance of SVMs learned using eigengenes as opposed to differentially expressed genes (Table 3).

We combined coexpression network analysis with Bayesian networks to model the interactions between thousands of genes in one network. Our model delineates the association between gene modules and the disease type (Fig. 3, Supplementary File S2, and Table 4).

### Comparison with other predictive models

We used the learned Bayesian network as a predictive model. Our results show that this model outperforms the margin tree classifier that Mills *et al*. fitted to the MILE microarray dataset^30^. To the best of our knowledge, Mills *et al*. is the only other group that has performed a similar study on this dataset. Their study involved a more complicated task, because it aimed at classifying 18 types of myeloid malignancies. Among all these types, they reported that their classifier had the least recall for MDS (i.e., 50%). Because the performance of margin trees on microarray data is similar to the nearest centroids^32^, we expect that our predictive model will also outperform the nearest centroids. Furthermore, evaluating our predictive model in an independent RNA-Seq dataset (BCCA) ensured that our network analysis is robust with respect to the underlying profiling technology (Table 2)^58^.

The accuracy of the SVM model substantially increases from 68% to 89% when, instead of individual genes, we use the eigengenes obtained from the gene network (Table 3 and Fig. 4). This shows that eigengenes are more informative and robust compared to individual differentially expressed genes, and that they can be useful and preferable for the analysis and comparison of expression profiles. When eigengenes are used as features in both predictive models, SVM has a similar accuracy compared to BN (89%), a slightly higher precision (92% vs. 88%), and a lower recall (92% vs. 98%). For clinical usage, the choice between these two approaches will depend on the preference between precision and recall. While we were writing this paper, we published Foroushani *et al*.^57^, which is focused on the biological significance of the gene modules. In that paper, we reported that a simple decision tree has a high accuracy on the MILE and BCCA datasets, which once again underlines the relatively high predictive power of eigengenes.

Our methodology is different from other BN approaches in which each random variable represents the expression of a *single* gene^9–15^. Because the number of training samples in real applications is usually limited to a few hundred cases, those approaches are not generally suitable to model many (thousands of) genes in a Bayesian network. One workaround is to filter genes before BN learning (e.g.^8,29^, and also the dedup function in the *bnlearn* package), which is inefficient due to information loss. The genes in a gene module are either highly correlated or highly anticorrelated, and they generally contribute to the same biological processes. Therefore, we do not model the probabilistic dependencies between the genes *within a module*, unlike the modular network learning approach^59,60^, which can unnecessarily complicate learning the structure of the BN. Although we compute the eigengene of a gene module using PCA, our approach is fundamentally different from *applying PCA directly to the entire expression profile*. PCA does not preserve the modular structure, and this can result in substantial and undesirable loss of information^61^. We implemented one of many ways in which a Bayesian network could be designed, trained, and used to infer information from eigengenes. We discuss some of the prominent alternative approaches in Supplementary Note S1.

### Limitations

One limitation of the employed coexpression network analysis is that each gene can be a member of only one module. This is suboptimal because some gene products can contribute to multiple biological processes. Fuzzy or soft clustering techniques^62,63^ and the incorporation of prior biological knowledge from pathway databases^64–66^ may address this challenge. Our predictive model is based on the expression of thousands of genes. The large number of genes makes our test difficult to apply in clinical settings. This can be addressed by excluding the genes that have a relatively smaller contribution to the eigengenes. Specifically, a greedy algorithm can be used to exclude the genes that have a smaller absolute weight (loading)^67^.

It would be interesting to reverse the train-test datasets and evaluate the performance of a model trained on RNA-Seq data. However, the current BCCA dataset has limited samples, so we leave this experiment for future work. We chose the MILE and BCCA datasets based on the following criteria: (1) Each dataset includes samples from both AML-NK and MDS cases, and (2) the number of cases of each disease is at least 20. To the best of our knowledge, the MILE and BCCA datasets are currently the only available datasets that meet these criteria. A couple of related datasets are available, including GSE34860^68^, GSE12417^69^, Leucegene (https://leucegene.ca), and The Cancer Genome Atlas (TCGA)^70^ with 78, 242, 46, 74 AML-NK cases, respectively. However, these datasets do not include MDS samples; therefore, they could not be used for the validation of our binary classifier. Combining these datasets with the MDS samples from other datasets could be problematic because of possible batch effects^71^. Also, the expression profile of 159 MDS cases in the GSE58831 dataset^72^ could not be directly compared to the MDS samples analyzed in this study because of the difference in the tissues. Specifically, the former is a gene expression profile of only CD34+ cells, while the BCCA and MILE datasets are based on whole peripheral blood samples.

### Generalizations and future directions

In this study, we developed and applied a new method to a solve a *classification* problem (i.,e., supervised learning), namely, distinguishing MDS from AML. Our classifier showed a remarkable performance although MDS is reported to be a heterogeneous disease. For example, the goal of Mills *et al*. study was to identify subtypes of MDS and determine the prognosis of each subtype^30^. This goal can be formulated as a *clustering* problem (i.e.,unsupervised learning). We anticipate that eigengenes can be useful to achieve this goal. For example, a *hidden* discrete random variable can be added to the Bayesian network structure to model the disease subtype.

Our model identified gene modules that are associated with the disease type. Some of these modules overrepresent genes that are related to particular biological pathways, including the extracellular matrix and homeobox genes, as we described elsewhere^57^. Future functional analysis can determine the role of the corresponding genes in myeloid malignancies and explain why these features (biomarkers) can differentiate between the two diseases with relatively high accuracy.

This study illustrates the potential of our approach, which scales up network analysis to thousands of genes. Our methodology can be useful in studying other diseases using existing datasets. The results of such experiments will be useful in pinpointing the cause and molecular mechanisms of diseases.

## Conclusion

Network analysis is useful for extracting informative biomarkers (features) from gene expression profiles. In particular, we showed that eigengenes have more predictive power than individual genes. A Bayesian network can be fitted to these data to model the association between the gene modules and the biological, or clinical, condition of interest. We compare our classifier with a support vector machine (SVM), which shows that the strength of our approach lies in the way we employ eigengenes as biological signatures (i.e., features). The SVM preforms unsatisfactory when we use individual genes as features, but when we use eigengenes as features, it has a performance comparable to the Bayesian network. Nevertheless, our Bayesian network approach is advantageous because it readily delineates the features that are most associated with the disease type, unlike SVM, which is more a black box classifier.

## Methods

### Ethics approval and consent to participate

This study was approved by the University of British Columbia–British Columbia Cancer Agency Research Ethics Board (UBC–BCCA REB) under protocol H13-02687 “Genomic analysis of molecular changes in myeloid malignancy”. The informed consent of participants was provided before specimen acquisition under the guidelines of the Leukemia/Bone Marrow Transplant Program at Vancouver General Hospital, as approved by the UBC–BCCA REB (protocol H04-61292). For historically-banked anonymized specimens (i.e., Legacy cell bank specimens), a waiver of consent was provided by the UBC–BCCA REB (protocol H09-01779). This protocol states: Genomic data obtained from these samples may be posted on access restricted sites as required for publications. This is covered by transfer contracts governed by our Technology Development Office. Transfer of material outside the institution would also be covered by Material Transfer Agreements (MTAs).

### The MILE dataset

We used the GEO2R tool^73^ to download gene expression data from the Gene Expression Omnibus (GEO) repository. We downloaded this dataset with accession number GSE15061, which is part of the MILE series (i.e., $\underline{{\rm{m}}}{\rm{icroarray}}\,\underline{{\rm{i}}}{\rm{nnovations}}\,\underline{{\rm{i}}}{\rm{n}}\,\underline{{\rm{le}}}{\rm{ukemia}}$). It consists of 164 MDS samples as well as 202 AML samples^30^, where 181 AML samples have normal karyotypes (AML-NK) and the remaining 21 AML samples have complex aberrant karyotypes.

We used the *limma* package (Version 3.28.5) to compute a p–value for each probe with a moderatet t-test^74^. The null hypothesis was that the probe was expressed the same in AML and MDS. We sorted the probes based on their p–values (i.e., variation across disease types). Consistent with the approach taken by other scholars in applying gene network analysis^10,75^, we kept all of the top third of the most variably expressed probes (*n* = 18,200) in our study. We used Custom CDF^76^ (Version 15) to map probes to Entrez-gene IDs. This mapping was not one-to-one, and we used the following approach to project the data from the probe level to the gene level: First, we excluded probes that were mapped to multiple Entrez-gene IDs. Out of 18, 200 probes, 13, 294 remained. Next, among all probes that were mapped to a specific Entrez-gene, the probe with the lowest p–value was chosen as the *representative* of that gene. That is, we considered the most differentially expressed probe as the representative of a gene. Based on our previous experiments^57^, we preferred this approach to alternative approaches such as using the mean or median of the expression of probes. Multiple probes that map to the same gene may measure the expression of different transcripts^77^. The alternative approaches can introduce redundant noise into the analysis when, for example, only one of these transcripts is differentially expressed. Also, if a transcript is upregulated and another transcript of the same gene is downregulated, then computing the average expression over probes could result in missing the potential relevance of the gene to the disease. If a gene had only one corresponding probe, that single probe was taken as the representative of the gene.

Our approach resulted in an expression profile consisting of 9,166 probes, where each of these probes represented a unique Entrez-gene with expression values for 202 AML cases and 164 MDS cases. We stored these data in two 9,166 × 202 and 9,166 × 164 matrices. We used these data as training data to identify gene modules and to learn the BN structure. We also compared the predictive value of these 9,166 genes with a shorter list of differentially expressed genes.

### The BCCA dataset

We used RNA sequencing dataacquired at the British Columbia Cancer Agency (BCCA) as the test data to evaluate the performance of our predictive model. This dataset contains 54 AML-NK and 22 MDS samples (peripheral blood cells or bone marrow blast). As detailed in the Supplementary File S5, we mapped the reads using Sailfish to quantify the gene expression and to compute RPKM values (i.e., $\underline{{\rm{r}}}{\rm{eads}}\,\underline{{\rm{p}}}{\rm{er}}\,\underline{{\rm{k}}}{\rm{ilobase}}\,{\rm{of}}\,{\rm{transcript}}\,{\rm{per}}\,\underline{{\rm{m}}}{\rm{illion}}\,{\rm{mapped}}\,{\rm{reads}}$)^78^. We inferred the eigengenes in the BCCA dataset using the logarithm of RPKM values in base 10 and the project.eigen function of the *Pigengene* package (https://bioconductor.org/packages/Pigengenehttps Version 1.2.0).

The eigengenes are available in Supplementary File S1. Because BCCA dataset was the test dataset, we did not use it in the module identification and BN learning procedures.

### Identifying gene modules

We used the R^79^ package *WGCNA*^5^ (Version 1.41) to perform coexpression network analysis on the 202 AML samples from the MILE dataset. *WGCNA* defines the similarity between two genes as the absolute value of the Pearson correlation of their expression levels. Using the pickSoftThreshold function with the default parameters, the power (*β*) parameter was inferred to be 8. For every integer *β* value in the range of 1–20, this function raises the similarity between all gene pairs to *β*, then computes the scale-free topology fit value of the resulting network^80^. The smallest *β* that results in a fit value more than RsquaredCut which is 0.85 by default, is returned as the suggested power parameter (Supplementary Fig. S5).

We used the blockwiseModules () function to identify gene modules based on the Pearson correlation of their expression. For better results, we set the parameter maxBlockSize = 9166 so that the process was performed in only one block. This prevented errors that could have occurred when merging the results from smaller blocks. We set TOMType = “unsigned”, and we used the default values for the rest of the arguments of blockwiseModules ().

*WGCNA* identified 33 modules. The largest and smallest modules consisted of 888 and 21 genes, respectively. Module sizes had a mean, median, and standard deviation of 153, 75, and 188, respectively (Supplementary Fig. S1). These modules were relatively stable with respect to the number of analyzed genes. That is, when we used 99%, 98%, 97%, 96%, 95%, 90%, and 80% of the 9,166 genes, the resulting modules had relatively high overlaps with the 33 modules that were identified using all the 9,166 genes (Supplementary Table S1). *WGCNA* could not confidently assign 4,125 genes to any of the modules because they showed little correlation with any other gene. These uncorrelated genes were designated as module 0 and were excluded from the rest of the analysis.

### Computing eigengenes

An eigengene of a module is a weighted average of the expressions of all the genes in that module. These weights are adjusted so that the loss in the biological information is minimized^7,81^. To compute eigengenes, we used principal component analysis (PCA)^81^, similar to the approach developed by Oldman *et al*. but with the following oversampling modification: We balanced the number of AML and MDS cases in the MILE dataset using oversampling, so that both disease types had comparable representatives in the analysis. That is, we repeated the data of each AML and MDS case 9 and 11 times, respectively. This resulted in 1,818 AML samples and 1,804 MDS samples. Then, we applied the moduleEigengenes () function from the *WGCNA* package to the oversampled data. This function computed the first principal component of each module, which maximized the explained variance, thus ensuring the loss in the biological information was minimized (Supplementary File S1)^7,81^.

### Designing the Bayesian network structure

Each of the 33 eigengenes corresponds to one observed random variable in our BN. In addition, the network has one binary variable, *Effect*, which models the disease type^82^. *Effect* is observed during the training phase. It is equal to 1 for AML, and it is 0 otherwise. By construction, no node is allowed to be a child of *Effect*. This design simplifies the inference using *bnlearn*; however, it may not be optimal for predicting the disease type, as we explain in the Discussion.

To implement the above property, we blacklisted all outgoing edges from the *Effect* node. The Markov property of BNs implies that, given its parents, the *Effect* node is independent from the rest of the network. More specifically, the Markov blanket of a node is a set of nodes that consists of the parents of the node, the children of the node, and any other parents of the children of that node^83^. In our model, the Markov blanket for the *Effect* node includes only its parents, because it does not have any children by construction. The Markov property of BNs then states that, conditioned on the Markov blanket of a node, the probability distribution of the node is independent from the rest of the network. In other words, the parents of the *Effect* node contain all the knowledge needed to predict its value. We inferred the value of *Effect* to predict the disease type in the test dataset.

### Subsampling

We did not used the BN shown in Fig. 3 to predict the disease type. Instead,we performed subsampling and predicted the disease type based on the majority vote of the individual models. That is, we randomly partitioned the training samples into five subsets that were almost equal in size. We fitted a BN using four of the five training subsets, and we repeated this procedure five times to obtain five Bayesian networks (Supplementary File S2). For each case in the test dataset, we inferred the value of *Effect* using these five BN models (Supplementary File S3). We considered the majority vote of five predictions as the ultimate prediction for each test case. Our subsampling approach is similar to the FeaLect methodology^40^, which roots in the statistical method developed by Politis and Romano^84,85^. This resampling approach is more generally applicable^86^ and slightly different from the common bootstrap aggregating (bagging) approach^87,88^ in that sampling is done without replacement^84,85^.

### Cross-validation

To assess the performance of the predictive model on the training dataset, we performed 5-fold cross validation (Supplementary Fig. S6). That is, we randomly partitioned the training samples into five subsets that were almost equal in size. We set aside one partition as the validation set, and we used the rest of samples to fit a BN. Using this BN, we inferred the value of *Effect* for each case in the validation dataset. In this way, the disease type of each training case is predicted by a BN that is trained using a subset of samples, which does not include that particular training case.

### Learning the BN structure and parameters

There are different approaches for fitting a BN to a dataset. A common approach is to optimize the Bayesian Dirichlet equivalent (BDe) scoring metric^89^. The BDe score is proportional to the posterior probability of a Bayesian network structure given the data and it has the *event equivalence* property. That is, two Bayesian network structures that represent the same set of independence assertions have equal BDe scores. A comparative study by Yu *et al*. (2002) using simulated data suggests that an appropriate Bayesian network inference approach to recover genetic pathways is to employ a greedy search method with random restarts to optimize BDe score, and to employ 3-interval hard discretization^90^. Accordingly, we discretized our eigengene expression data into three levels using Hartemink’s method^91^. We used the bn.boot() function from the *bnlearn* package (Version 4.0) to fit several BN structures (DAGs) to the discretized data^92^. This function used a hill-climbing strategy to optimize the BDe score^89^. Consistent with the approach taken by other scholars^10^, we averaged one-third of the networks with the highest scores to obtain the consensus network. We used the bn.fit function to fit the parameters (probability tables) of the consensus network. The diagram in Supplementary Fig. S2 shows these steps in more detail. The names of the functions, which we used from the *bnlearn* package, are highlighted in red.

The analysis of the BDe scores showed that learning 500 networks is enough to infer the consensus BN for our data (Supplementary Fig. S3). Accordingly, we chose to learn 500 networks for our experiments. Also, the consensus networks obtained from both 500 and 5,000 networks have similar structures (Fig. 3). For example, in both models, modules 6 and 12 are the parents of the *Effect* node, and module 9 has no children.

### Inference

We used the consensus model to predict the disease type. Specifically, we used the predict.bn.fit function of the *bnlearn* package and set method = bayes−lw to infer the value of the *Effect* node. With this setting, this function uses the likelihood weighting algorithm^93,94^ to estimate the value for the *Effect* node that has the highest probability conditioned on the observed eigengene data. Because *Effect* has no children by construction, its Markov blanket consists of only its parents, which are all observed random variables. Therefore, in this specific case, the above approach is equivalent to identifying the value for *Effect* that is most probable according to the conditional probability table for this node.

### Measuring the performance of the predictive model

To assess the performance of our predictions, we considered AML cases as positive samples and considered MDS cases as negative samples. We computed several statistical measures of the performance^95^, including (a) accuracy, which is the proportion of correctly predicted samples among all predictions, (b) sensitivity, also known as recall, which is the ratio of correctly predicted positive samples over all positive samples, (c) precision, also known as positive predictive value, which is the ratio of correctly predicted positive samples over all predicted positive samples^96,97^, and (d) AUC, which is the area under the receiver operating characteristic (ROC) curve^98^. We used the *caret* package (short for $\underline{c}lassification\,\underline{a}nd\,\underline{re}gression\,\underline{t}raining,$ Version 6.0–76) to calculate the performance of our predictions^95^. We used the plot.ROC function from the MCLSLLreproduction package to plot several ROC curves in one frame^99^.

### Breast cancer datasets

The METABRIC discovery and validation datasets are available from the European Genome-phenome Archive with the study accession number EGAS00000000083. We applied the *Pigengene* package on the METABRIC discovery dataset to identify 16 modules, and to compute the corresponding eigengenes (Supplementary File S6). The rest of the analysis on the breast cancer data was similar to the methodology that we developed to analyze leukemia, which is described in this paper.

### Data availability

All data analysed during this study are included in the Supplementary Information files, which can be used to reproduced the results.

## Electronic supplementary material

Supplementary Figures

Supplementary File S1

Supplementary File S2

Supplementary File S3

Supplementary file S4

Supplementary file S5

Supplementary File S6.

Supplementary Table S1

Supplementary Table S2.

Supplementary Note S1

Supplementary File descriptions
