# Supplementary material for: Applications of Bayesian network models in predicting types of hematological malignancies
Source: Sci Rep. 2018 May 3;8:6951. doi: 10.1038/s41598-018-24758-5 (PMC5934387; doi:10.1038/s41598-018-24758-5)
Supplement: Supplementary file 1 — Supplementary Figures [file 41598_2018_24758_MOESM1_ESM.pdf]

Applications of Bayesian network models in predicting types of hematological malignancies

Rupesh Agrahari[1,+], Amir Foroushani[1,+], Thomas Roderick Docking[2], Linda Chang[2], Gerben Duns[2], Monika Hudoba[3], Aly Karsan[2,‡], and Habil Zare[2,‡,*]

[1]Department of Computer Science, Texas State University, San Marcos, Texas, 78666, USA
[2]Department of Pathology and Laboratory Medicine, British Columbia Cancer Agency, Vancouver, British Columbia, V5Z 4E6, Canada
[3]Department of Pathology and Laboratory Medicine, Vancouver General Hospital, Vancouver, British Columbia, V5Z 1M9, Canada
[*]zare@txstate.edu
[+]These authors contributed equally to this work.
[+]These senior authors contributed equally to this work.

List of Supplementary Figures

S1 The distribution of module sizes. 2
S2 Graphical presentation of the steps for learning the BN structure using the *bn-learn* package. 3
S3 Score improvement. 4
S4 Expression of the 33 top differentially expressed genes on the MILE and BCCA dataset. 5
S5 The scale-free topology values. 6
S6 Graphical presentation of the steps for performing cross-validation on the training (MILE) dataset. 7

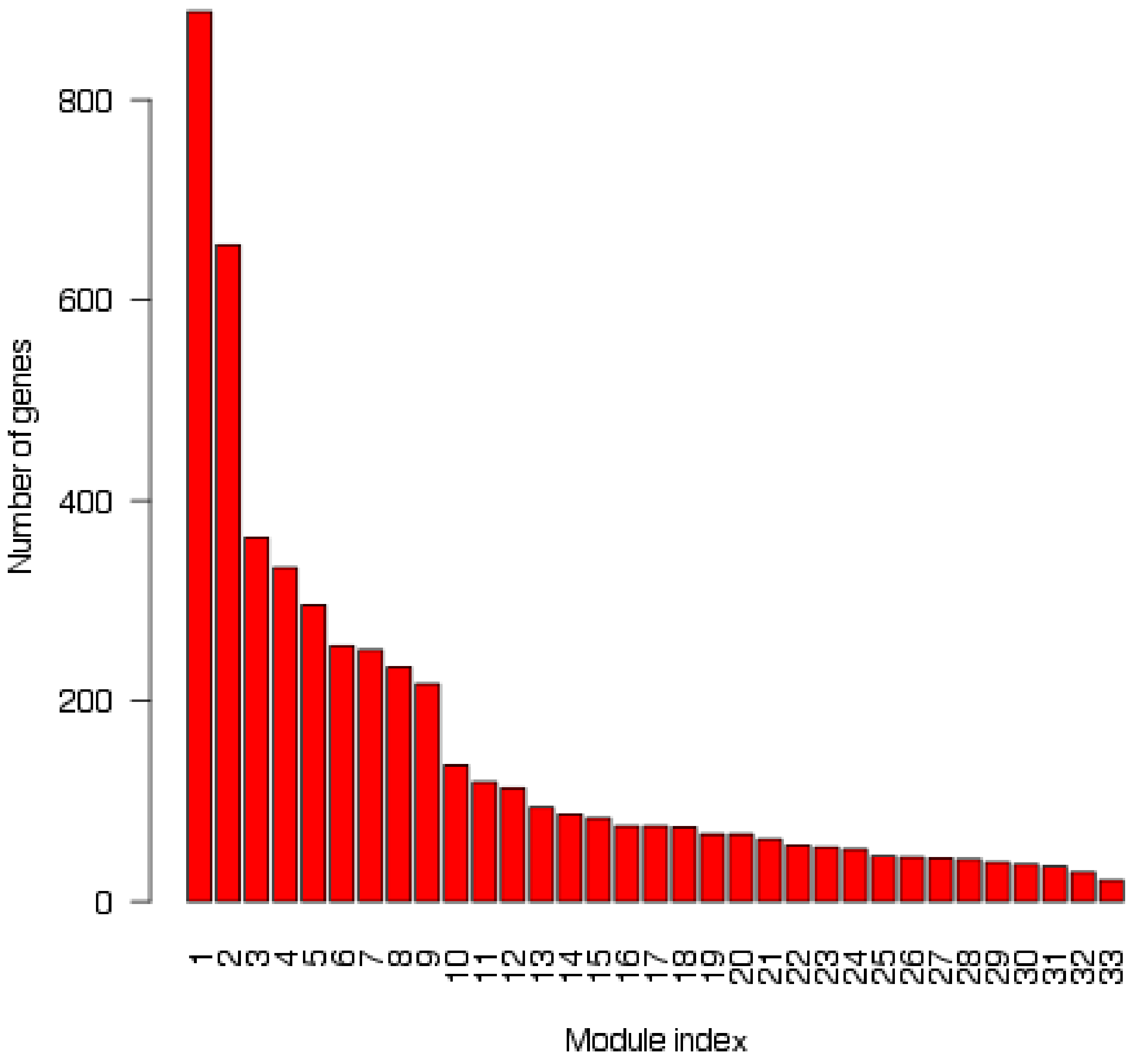

Supplementary Figure S1. The distribution of module sizes.

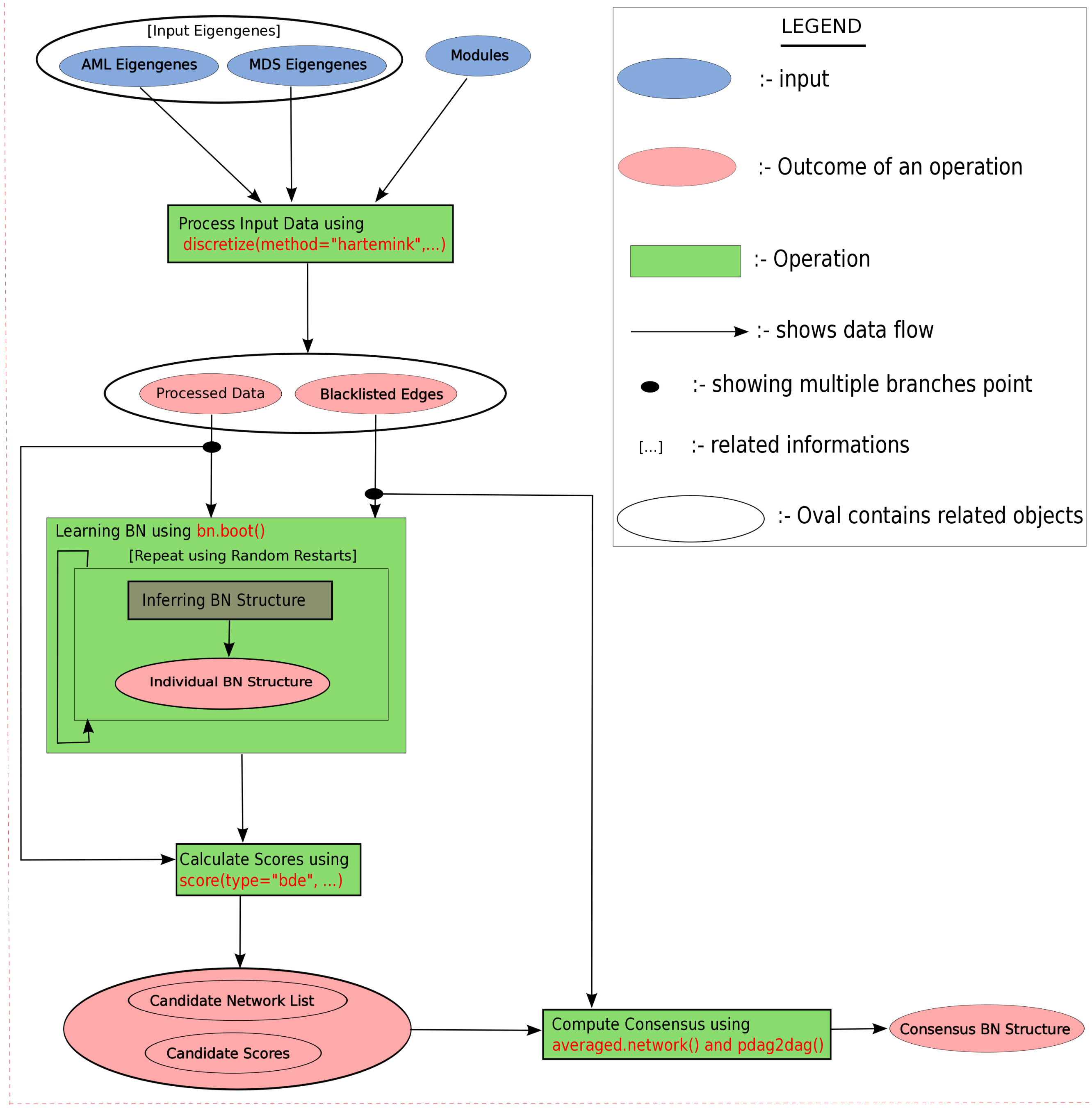

Supplementary Figure S2. Graphical presentation of the steps for learning the BN structure using the *bnlearn* package. Observed random variables (input data) are the eigengene values obtained from the training (MILE) dataset. Eigengenes are discretized using Hartemink's method (the `discretize` function). The discretized eigengenes were used to learn 500 BNs with random restarts (the `bn.boot` function). The BDe scores are calculated for all learned BNs (the `score` function). The consensus network is inferred based on the top third networks with the best scores (the `averaged.network` and `pdag2dag` functions).

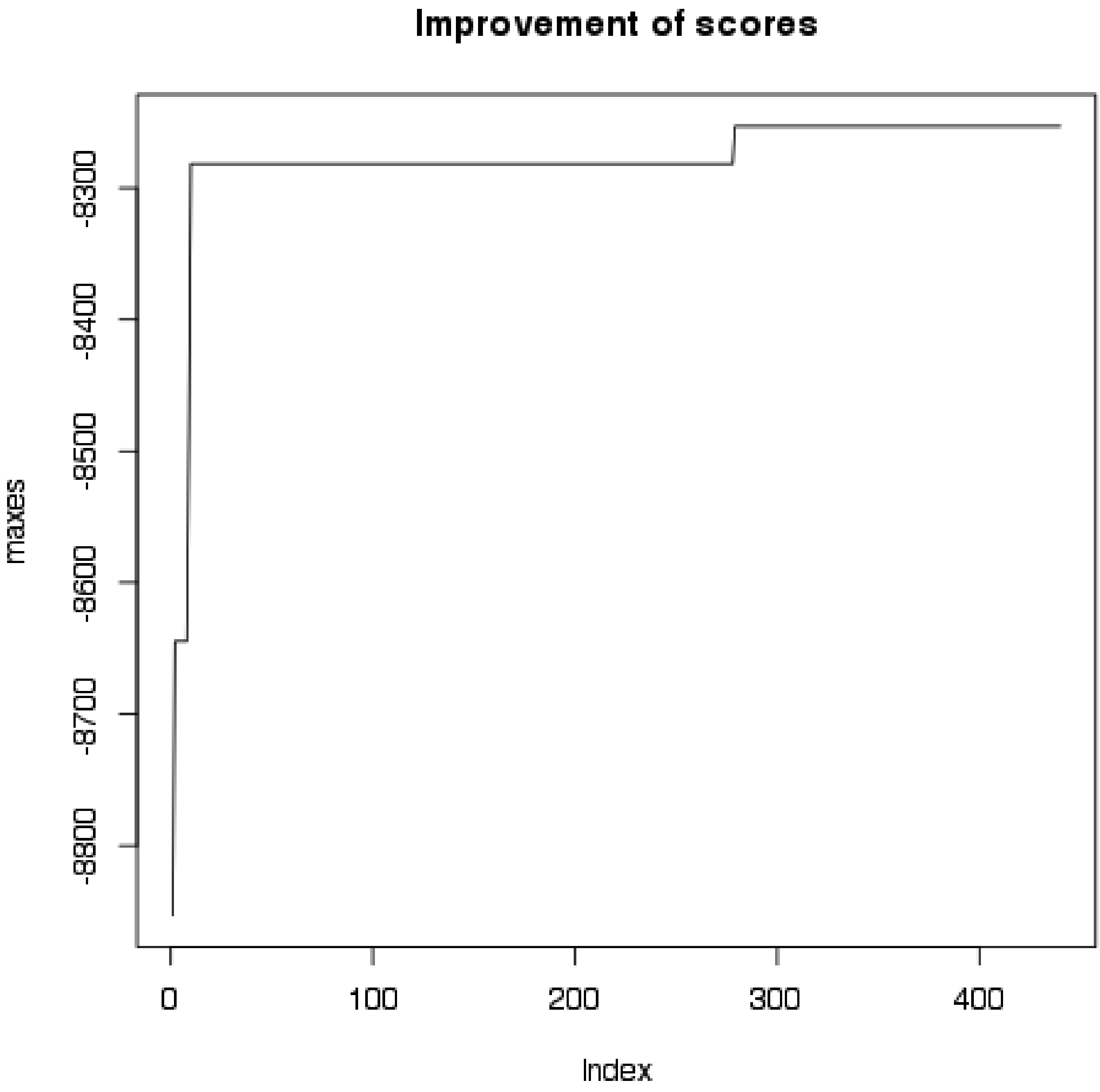

Supplementary Figure S3. Score improvement. For any number of learned BNs in the range of 1 to 500 (the x-axis), the BDe score of the best BN is shown on the y-axis. Scores did not improve beyond 300 networks.

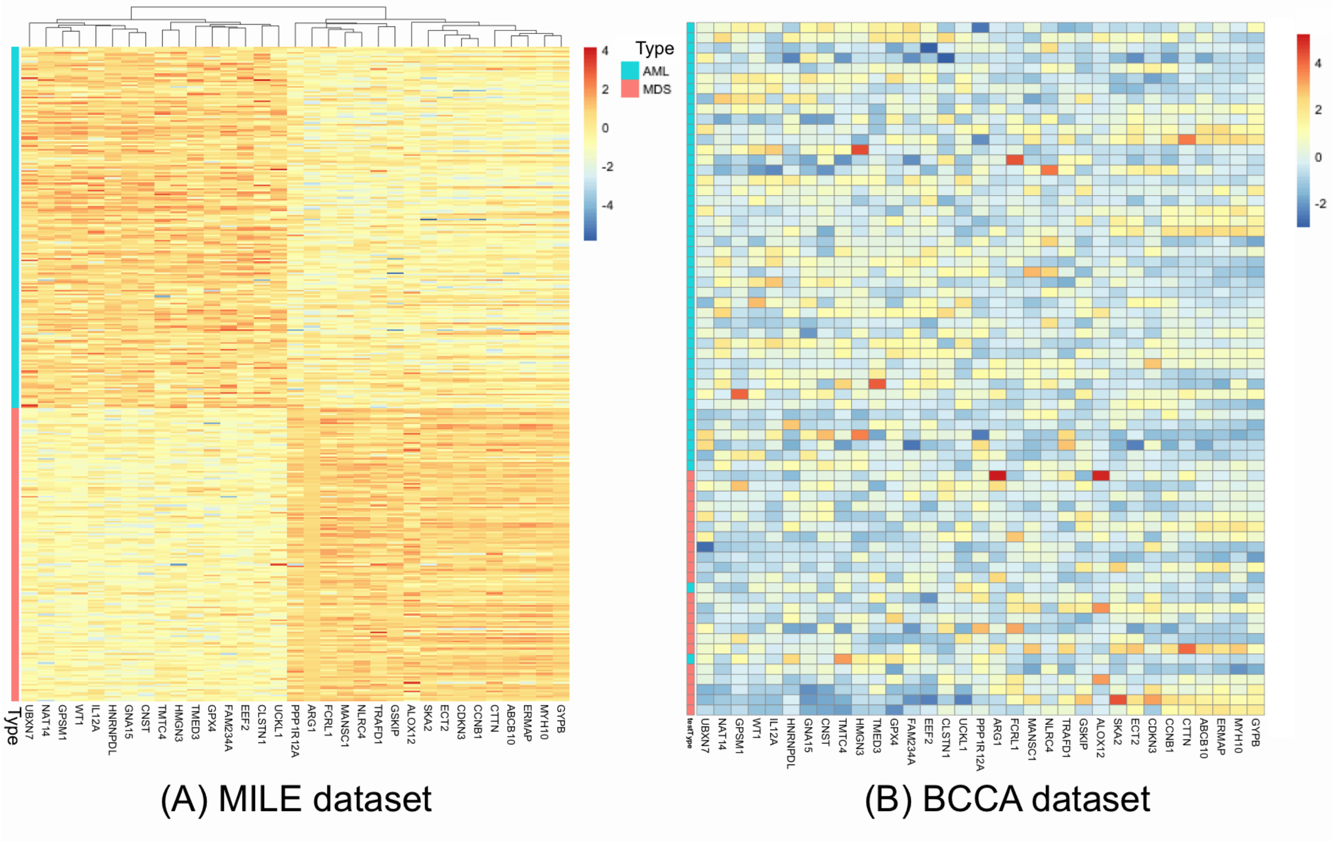

Supplementary Figure S4. Expression of the 33 top differentially expressed genes on the MILE and BCCA dataset. These genes are clearly differentially expressed in the MILE dataset **(A)** but not in the BCCA dataset **(B)**. This illustrates the normalization and standardization challenges in comparing the microarray and RNA-seq data, and highlights the significance of eigengenes as robust features with respect to the profiling platform.

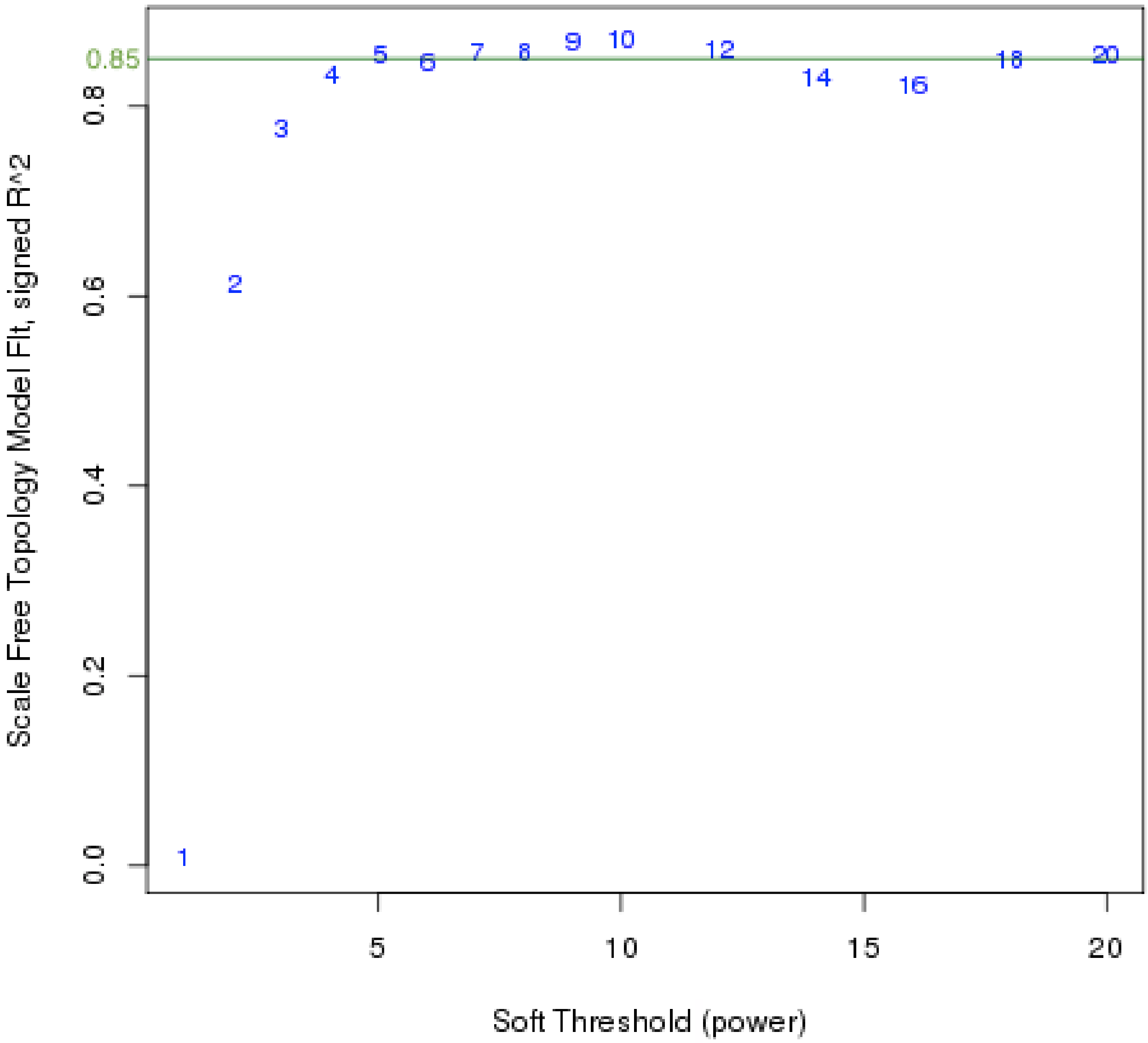

Supplementary Figure S5. The scale-free topology values.

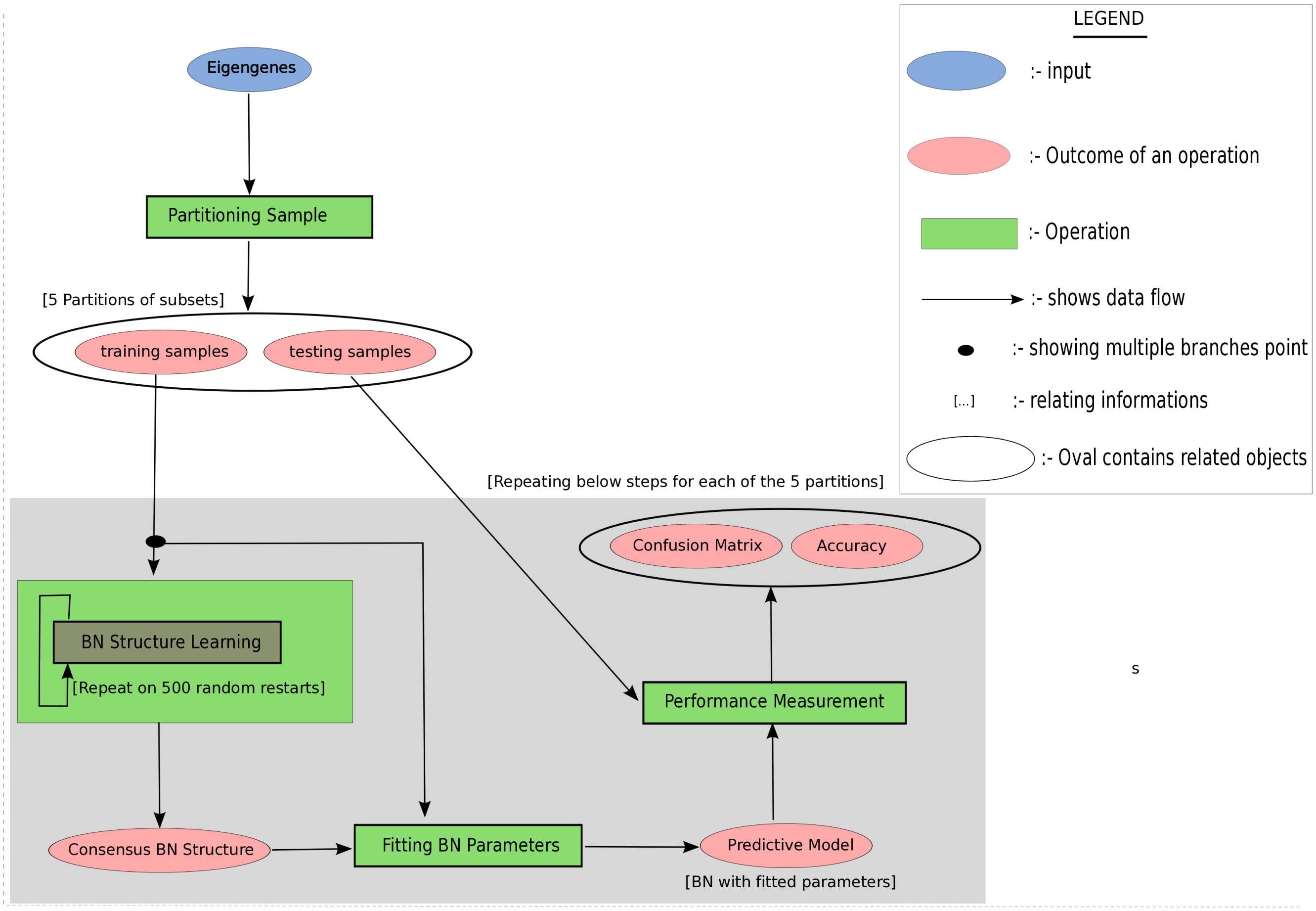

Supplementary Figure S6. Graphical presentation of the steps for performing cross-validation on the training (MILE) dataset.
