# Supplementary material for: Applications of Bayesian network models in predicting types of hematological malignancies
Source: Sci Rep. 2018 May 3;8:6951. doi: 10.1038/s41598-018-24758-5 (PMC5934387; doi:10.1038/s41598-018-24758-5)
Supplement: Supplementary file 3 — Supplementary File S2 [file 41598_2018_24758_MOESM3_ESM.pdf]

Applications of Bayesian network models in predicting types of hematological malignancies

Rupesh Agrahari[1,+], Amir Foroushani[1,+], Thomas Roderick Docking[2], Linda Chang[2], Gerben Duns[2], Monika Hudoba[3], Aly Karsan[2,‡], and Habil Zare[2,‡,*]

[1]Department of Computer Science, Texas State University, San Marcos, Texas, 78666, USA
[2]Department of Pathology and Laboratory Medicine, British Columbia Cancer Agency, Vancouver, British Columbia, V5Z 4E6, Canada
[3]Department of Pathology and Laboratory Medicine, Vancouver General Hospital, Vancouver, British Columbia, V5Z 1M9, Canada
[*]zare@txstate.edu
[+]These authors contributed equally to this work.
[+]These senior authors contributed equally to this work.

Supplementary File 2: The BN structures obtained from subsampling

The following figures show BN structures learned using eigengenes in the MILE dataset for each of the 5 subsamples. Each BN is the consensus network obtained from the top third of 500 fitted networks. The nodes are colored similar to Fig. 2 in the paper.

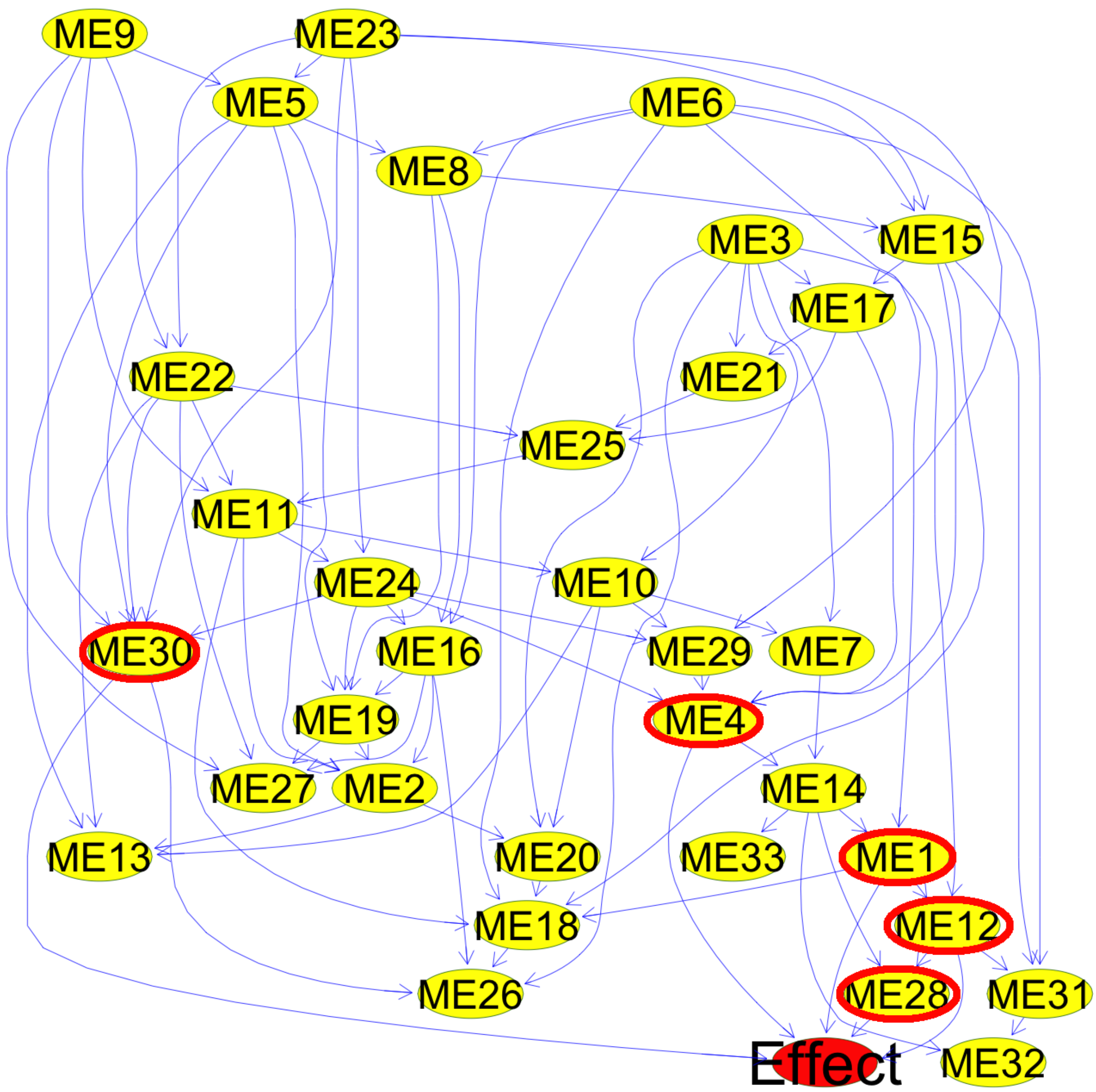
ME9
ME23
ME5
ME6
ME8
ME3
ME15
ME17
ME22
ME21
ME25
ME11
ME24
ME10
ME30
ME16
ME29
ME7
ME19
ME4
ME27
ME2
ME14
ME13
ME20
ME33
ME1
ME18
ME12
ME26
ME28
ME31
Effect
ME32

Figure 1

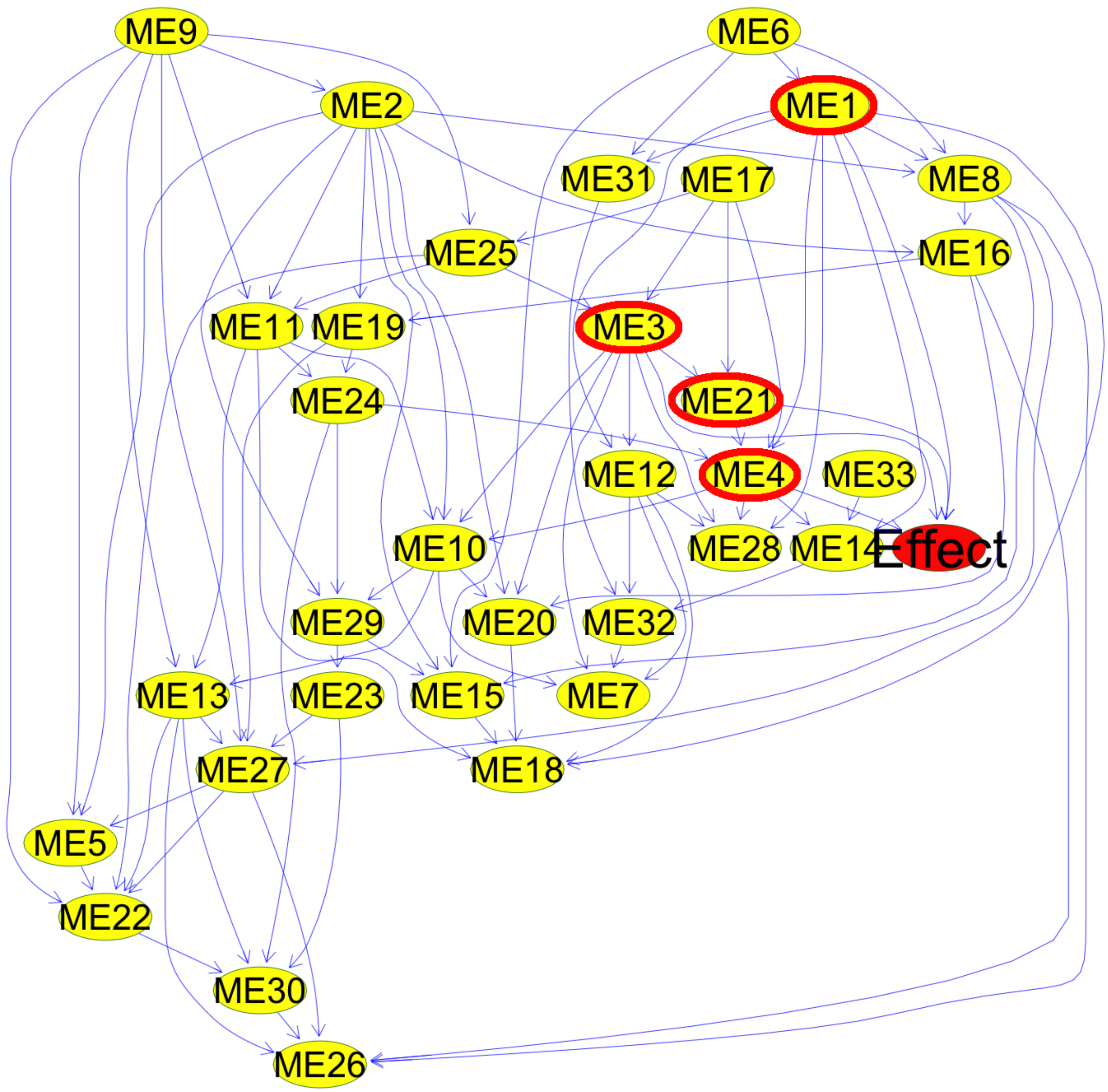
ME9
ME6
ME2
ME1
ME31
ME17
ME8
ME25
ME16
ME11
ME19
ME3
ME24
ME21
ME12
ME4
ME33
ME10
ME28
ME14
Effect
ME29
ME20
ME32
ME13
ME23
ME15
ME7
ME27
ME18
ME5
ME22
ME30
ME26

Figure 2

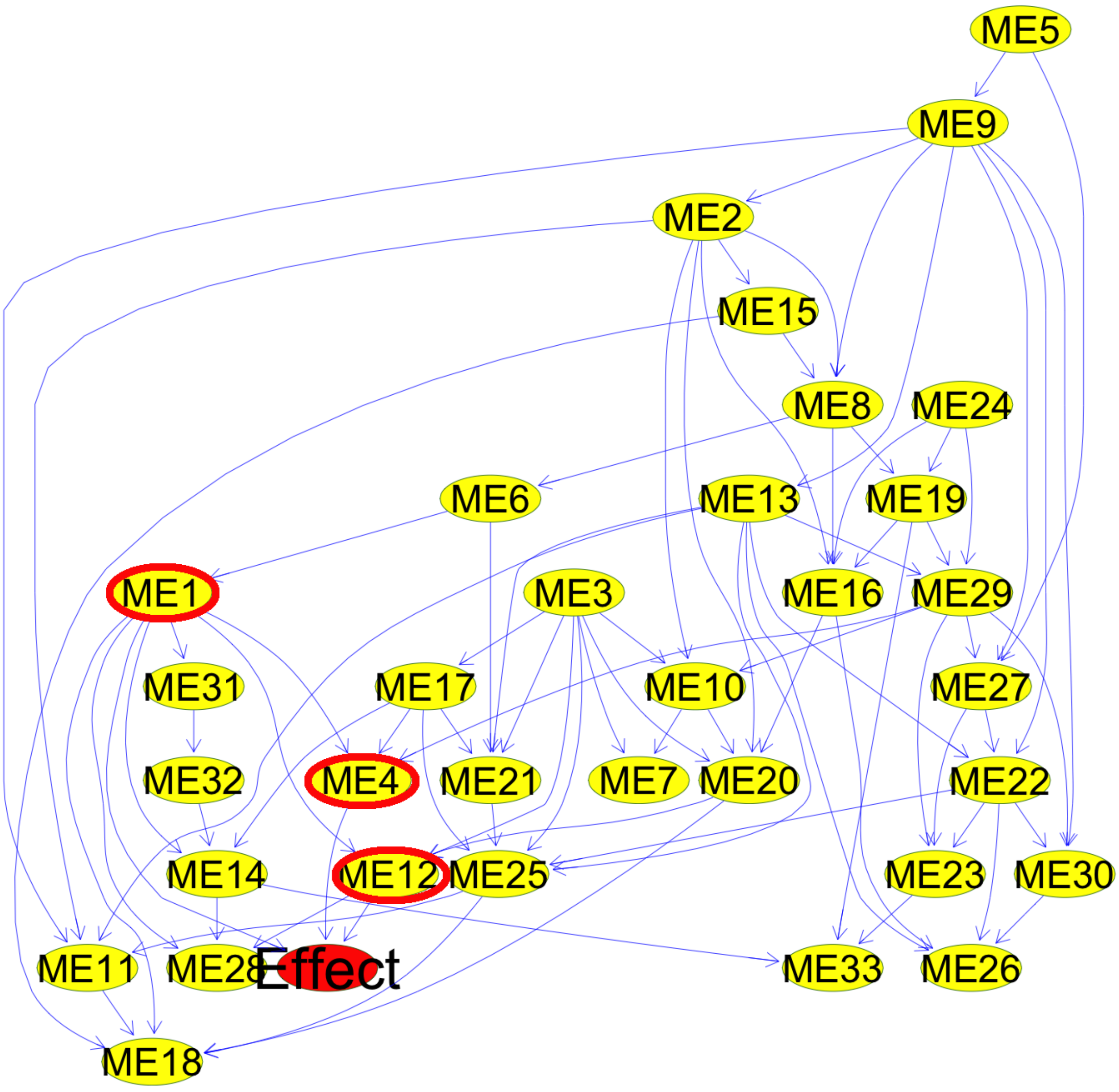
ME5
ME9
ME2
ME15
ME8
ME24
ME6
ME13
ME19
ME1
ME3
ME16
ME29
ME31
ME17
ME10
ME27
ME32
ME4
ME21
ME7
ME20
ME22
ME14
ME12
ME25
ME23
ME30
ME11
ME28
Effect
ME33
ME26
ME18

Figure 3

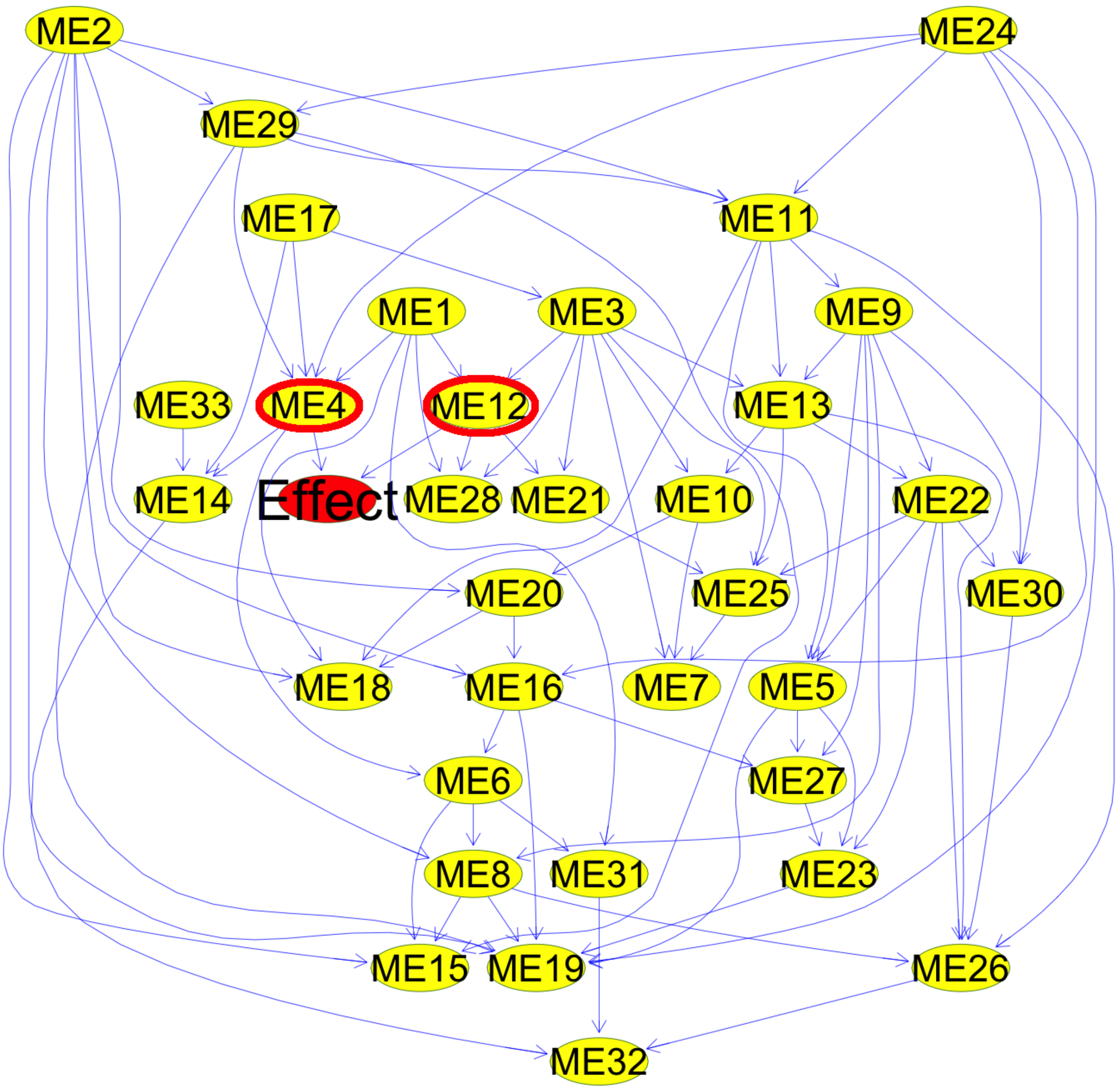
ME2
ME24
ME29
ME17
ME11
ME1
ME3
ME9
ME33
ME4
ME12
ME13
ME14
Effect
ME28
ME21
ME10
ME22
ME20
ME25
ME30
ME18
ME16
ME7
ME5
ME6
ME27
ME8
ME31
ME23
ME15
ME19
ME26
ME32

Figure 4

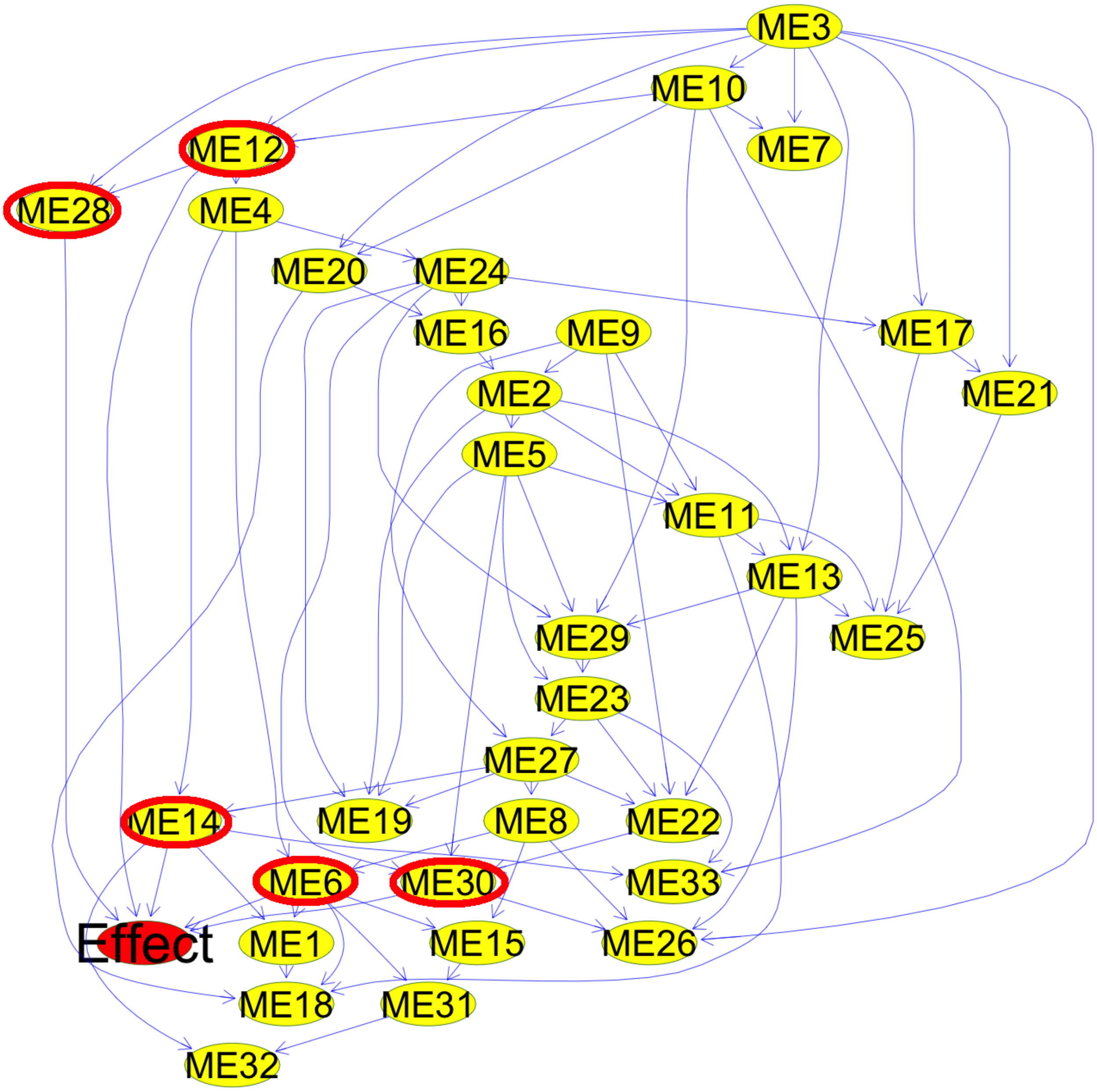
ME3
ME10
ME7
ME12
ME28
ME4
ME20
ME24
ME16
ME9
ME17
ME2
ME21
ME5
ME11
ME13
ME29
ME25
ME23
ME27
ME14
ME19
ME8
ME22
ME6
ME30
ME33
Effect
ME1
ME15
ME26
ME18
ME31
ME32

Figure 5
