# Supplementary material for: Applications of Bayesian network models in predicting types of hematological malignancies
Source: Sci Rep. 2018 May 3;8:6951. doi: 10.1038/s41598-018-24758-5 (PMC5934387; doi:10.1038/s41598-018-24758-5)
Supplement: Supplementary file 5 — Supplementary file S4 [file 41598_2018_24758_MOESM5_ESM.pdf]

Applications of Bayesian network models in predicting types of hematological malignancies

Rupesh Agrahari[1,+], Amir Foroushani[1,+], Thomas Roderick Docking[2], Linda Chang[2], Gerben Duns[2], Monika Hudoba[3], Aly Karsan[2,‡], and Habil Zare[2,‡,*]

[1]Department of Computer Science, Texas State University, San Marcos, Texas, 78666, USA
[2]Department of Pathology and Laboratory Medicine, British Columbia Cancer Agency, Vancouver, British Columbia, V5Z 4E6, Canada
[3]Department of Pathology and Laboratory Medicine, Vancouver General Hospital, Vancouver, British Columbia, V5Z 1M9, Canada
[*]zare@txstate.edu
[+]These authors contributed equally to this work.
[+]These senior authors contributed equally to this work.

Supplementary File 4: The confusion matrices and performance of individual models obtained from 5 subsamples.

```
Partition 1: Confusion matrix and statistics

           Actual
Prediction AML  MDS
       AML 184   7
       MDS  18  157

              Accuracy : 0.9317                    95% CI : (0.9008, 0.9553)
    No Information Rate : 0.5519          P-Value [Acc > NIR] : <2e-16

                 Kappa : 0.8628          Mcnemar's Test P-Value : 0.0455

           Sensitivity : 0.9109                Specificity : 0.9573
        Pos Pred Value : 0.9634             Neg Pred Value : 0.8971
            Prevalence : 0.5519             Detection Rate : 0.5027
   Detection Prevalence : 0.5219          Balanced Accuracy : 0.9341

       'Positive' Class : AML
```

Figure 1. **Performance of model 1 on the MILE (training) dataset**

```
Partition 2: Confusion matrix and statistics

          Actual
Prediction AML MDS
       AML 179   6
       MDS  23 158

               Accuracy : 0.9208                    95% CI : (0.8882, 0.9463)
    No Information Rate : 0.5519       P-Value [Acc > NIR] : < 2.2e-16

                  Kappa : 0.8414      Mcnemar's Test P-Value : 0.002967

            Sensitivity : 0.8861               Specificity : 0.9634
         Pos Pred Value : 0.9676            Neg Pred Value : 0.8729
             Prevalence : 0.5519            Detection Rate : 0.4891
   Detection Prevalence : 0.5055         Balanced Accuracy : 0.9248

       'Positive' Class : AML
```

Figure 2. **Performance of model 2 on the MILE (training) dataset**

```
Partition 3: Confusion matrix and statistics

          Actual
Prediction AML MDS
       AML 176   9
       MDS  26 155

               Accuracy : 0.9044                    95% CI : (0.8695, 0.9325)
    No Information Rate : 0.5519       P-Value [Acc > NIR] : < 2.2e-16

                  Kappa : 0.8085      Mcnemar's Test P-Value : 0.006841

            Sensitivity : 0.8713               Specificity : 0.9451
         Pos Pred Value : 0.9514            Neg Pred Value : 0.8564
             Prevalence : 0.5519            Detection Rate : 0.4809
   Detection Prevalence : 0.5055         Balanced Accuracy : 0.9082

       'Positive' Class : AML
```

Figure 3. **Performance of model 3 on the MILE (training) dataset**

```
Partition 4: Confusion matrix and statistics

           Actual
Prediction AML MDS
       AML 173  10
       MDS  29 154

              Accuracy : 0.8934                     95% CI : (0.8572, 0.9231)
    No Information Rate : 0.5519         P-Value [Acc > NIR] : < 2.2e-16

                 Kappa : 0.7869          Mcnemar's Test P-Value : 0.003948

           Sensitivity : 0.8564                  Specificity : 0.9390
        Pos Pred Value : 0.9454               Neg Pred Value : 0.8415
            Prevalence : 0.5519               Detection Rate : 0.4727
   Detection Prevalence : 0.5000            Balanced Accuracy : 0.8977

       'Positive' Class : AML
```

Figure 4. Performance of model 4 on the MILE (training) dataset

```
Partition 5: Confusion matrix and statistics

           Actual
Prediction AML MDS
       AML 195   9
       MDS   7 155

              Accuracy : 0.9563                     95% CI : (0.93, 0.9748)
    No Information Rate : 0.5519         P-Value [Acc > NIR] : <2e-16

                 Kappa : 0.9115         Mcnemar's Test P-Value : 0.8026

           Sensitivity : 0.9653                  Specificity : 0.9451
        Pos Pred Value : 0.9559               Neg Pred Value : 0.9568
            Prevalence : 0.5519               Detection Rate : 0.5328
   Detection Prevalence : 0.5574            Balanced Accuracy : 0.9552

       'Positive' Class : AML
```

Figure 5. Performance of model 5 on the MILE (training) dataset

```
Confusion matrix and statistics on BCCA data using BN for partition 1:

          Actual
Prediction AML MDS
       AML  49   4
       MDS   3  18

               Accuracy : 0.9054             95% CI : (0.8148, 0.9611)
    No Information Rate : 0.7027             P-Value [Acc > NIR] : 2.607e-05

                  Kappa : 0.7706          Mcnemar's Test P-Value : 1

            Sensitivity : 0.9423                     Specificity : 0.8182
         Pos Pred Value : 0.9245                  Neg Pred Value : 0.8571
             Prevalence : 0.7027                  Detection Rate : 0.6622
   Detection Prevalence : 0.7162               Balanced Accuracy : 0.8802

       'Positive' Class : AML
```

Figure 6. Performance of model 1 on the BCCA (test) dataset

```
Confusion matrix and statistics on BCCA data using BN for partition 2:

          Actual
Prediction AML MDS
       AML  38   7
       MDS  14  15

               Accuracy : 0.7162            95% CI : (0.5995, 0.815)
    No Information Rate : 0.7027            P-Value [Acc > NIR] : 0.4562

                  Kappa : 0.3779         Mcnemar's Test P-Value : 0.1904

            Sensitivity : 0.7308                    Specificity : 0.6818
         Pos Pred Value : 0.8444                 Neg Pred Value : 0.5172
             Prevalence : 0.7027                 Detection Rate : 0.5135
   Detection Prevalence : 0.6081              Balanced Accuracy : 0.7063

       'Positive' Class : AML
```

Figure 7. Performance of model 2 on the BCCA (test) dataset

```
Confusion matrix and statistics on BCCA data using BN for partition 3:

          Actual
Prediction AML MDS
       AML  50   8
       MDS   2  14

               Accuracy : 0.8649              95% CI : (0.7655, 0.9332)
    No Information Rate : 0.7027              P-Value [Acc > NIR] : 0.0009218

                  Kappa : 0.649            Mcnemar's Test P-Value : 0.1138463

            Sensitivity : 0.9615                      Specificity : 0.6364
         Pos Pred Value : 0.8621                   Neg Pred Value : 0.8750
             Prevalence : 0.7027                   Detection Rate : 0.6757
   Detection Prevalence : 0.7838                Balanced Accuracy : 0.7990

       'Positive' Class : AML
```

Figure 8. **Performance of model 3 on the BCCA (test) dataset**

```
Confusion matrix and statistics on BCCA data using BN for partition 4:

          Actual
Prediction AML MDS
       AML  52   9
       MDS   0  13

               Accuracy : 0.8784              95% CI : (0.7816, 0.9429)
    No Information Rate : 0.7027              P-Value [Acc > NIR] : 0.0003169

                  Kappa : 0.67             Mcnemar's Test P-Value : 0.0076608

            Sensitivity : 1.0000                      Specificity : 0.5909
         Pos Pred Value : 0.8525                   Neg Pred Value : 1.0000
             Prevalence : 0.7027                   Detection Rate : 0.7027
   Detection Prevalence : 0.8243                Balanced Accuracy : 0.7955

       'Positive' Class : AML
```

Figure 9. **Performance of model 4 on the BCCA (test) dataset**

```
Confusion matrix and statistics on BCCA data using BN for partition 5:

          Actual
Prediction AML MDS
       AML  48   8
       MDS   4  14

               Accuracy : 0.8378             95% CI : (0.7339, 0.9133)
    No Information Rate : 0.7027             P-Value [Acc > NIR] : 0.005718

                  Kappa : 0.5904          Mcnemar's Test P-Value : 0.386476

            Sensitivity : 0.9231                     Specificity : 0.6364
         Pos Pred Value : 0.8571                  Neg Pred Value : 0.7778
             Prevalence : 0.7027                  Detection Rate : 0.6486
   Detection Prevalence : 0.7568               Balanced Accuracy : 0.7797

       'Positive' Class : AML
```

Figure 10. **Performance of model 5 on the BCCA (test) dataset**
