# Supplementary material for: Applications of Bayesian network models in predicting types of hematological malignancies
Source: Sci Rep. 2018 May 3;8:6951. doi: 10.1038/s41598-018-24758-5 (PMC5934387; doi:10.1038/s41598-018-24758-5)
Supplement: Supplementary file 6 — Supplementary file S5 [file 41598_2018_24758_MOESM6_ESM.pdf]

Applications of Bayesian network models in predicting types of hematological malignancies

Rupesh Agrahari[1,+], Amir Foroushani[1,+], Thomas Roderick Docking[2], Linda Chang[2], Gerben Duns[2], Monika Hudoba[3], Aly Karsan[2,‡], and Habil Zare[2,‡,*]

[1]Department of Computer Science, Texas State University, San Marcos, Texas, 78666, USA

[2]Department of Pathology and Laboratory Medicine, British Columbia Cancer Agency, Vancouver, British Columbia, V5Z 4E6, Canada

[3]Department of Pathology and Laboratory Medicine, Vancouver General Hospital, Vancouver, British Columbia, V5Z 1M9, Canada

[*] zare@txstate.edu

[+]These authors contributed equally to this work.

[+]These senior authors contributed equally to this work.

Supplementary File 5: RNA-Seq analysis on the BCCA dataset

We constructed a retrospective AML and MDS sample cohort with some known prior karyotype and genetic testing information. Samples were selected so as to encompass a broad range of myeloid malignancy subtypes. We used only AML-NK and MDS in this study.

Library Preparation and Sequencing

Sample Acquisition and Ethics

Peripheral blood and bone marrow samples were obtained from consenting patients via the Hematology Cell Bank of British Columbia (http://hematology.med.ubc.ca/research/hematology-cell-bank-of-bc/). Ethics protocols were all approved by the BCCA REB, under protocols H04-61292, H09-01779, H11-01484, and H13-02687.

RNA Extraction and Library Construction

RNA was manually extracted from bone marrow or peripheral blood using Qiagen Allprep kits. Total RNA samples were checked using Agilent Bioanalyzer RNA nanochip or Caliper GX HT RNA LabChip. Samples that passed quality check were arrayed into a 96-well plate. Following this, polyA+ RNA was purified using the 96-well MultiMACS mRNA isolation kit on the MultiMACS 96 separator (Miltenyi Biotec, Germany) from total RNA with on column DNaseI-treatment as per the manufacturer's instructions. The eluted polyA+ RNA was ethanol precipitated and resuspended in 10μL of DEPC treated water with 1:20 SuperaseIN (Life Technologies, USA).

Double-stranded cDNA was synthesized from the purified polyA+ RNA using the Maxima H Minus First Strand cDNA Synthesis Kit (Thermo Fisher Scientific Inc., USA) and random hexamer primers. Quality passed cDNA plate was fragmented by Covaris LE220 for 2x65 seconds at “Duty cycle” of 30%. The paired-end sequencing library was prepared following the BCCA Genome Sciences Centre paired-end library preparation plate based library construction protocol on a Biomek FX robot (Beckman-Coulter, USA). Briefly, the cDNA was subject to end-repair, and phosphorylation by T4 DNA polymerase, Klenow DNA Polymerase, and T4 polynucleotide kinase respectively in a single reaction, followed by cleanup using magnetic beads and 3’ A-tailing by Klenow fragment (3’ to 5’ exo minus). After cleanup, adapter ligation was performed. The adapter-ligated products were purified using magnetic beads, then UNG digested and PCR-amplified with Phusion DNA Polymerase (Thermo Fisher Scientific Inc., USA) using Illumina’s PE primer set in a single reaction, with cycle condition 37˚C 15min, 98˚C 1min followed by 13 cycles of 98˚C 15 sec, 65˚C 30 sec and 72˚C 30 sec, and then 72˚C 5min. The PCR products were purified and size selected using magnetic beads, checked with Caliper LabChip GX for DNA samples using the High Sensitivity Assay (PerkinElmer, Inc. USA) and quantified with the Quant-iT dsDNA HS Assay Kit using Qubit fluorometer (Invitrogen). Libraries were normalized and pooled. The final concentration was double checked and determined by Qubit dsDNA HS Assay for Illumina Sequencing.

Sequencing

For the first retrospective RNA-Seq cohort, we sequenced a single library per Illumina HiSeq 2000 lane, using 2x75bp reads, which resulted in approximately 400 million reads per library. 92 samples were initially submitted for library construction, of which 89 were successfully prepared and sequenced.

For the second retrospective RNA-Seq cohort, we sequenced two libraries per Illumina HiSeq 2000 lane, using 2x75bp reads, which resulted in approximately 200 million reads per library. 92 samples were initially submitted for library construction, of which 87 were successfully prepared and sequenced.

Bioinformatics Pipeline

Pipeline Overview

All samples were processed using customized in-house bioinformatics pipelines. The WGS and WES data was processed by the Bioinformatics Core at the BCCA Genome Sciences Centre, while the RNA-Seq data was processed by the Centre for Clinical Genomics Informatics group and the authors.

RNA-Seq Quality Control

We used RNA-SeQC (DeLuca et al., 2012) to gather quality metrics for the RNA-Seq libraries. This tool gathers standard sequence quality metrics, such as the count of uniquely aligned and duplicate reads, and RNA-specific metrics such as the proportion of rRNA reads, strandedness of

read alignments, and the proportion of reads aligning to exonic, intronic, intragenic, and intergenic regions.

Expression Quantification

Expression quantification was performed for all RNA-Seq and ssRNA-Seq libraries using sailfish version `0.9.0` (Patro, Mount, & Kingsford, 2014), using RefSeq gene models downloaded as GTF from the UCSC genome browser on 2014-08-21, with gene models from non-standard chromosome sequences removed. Both isoform- and gene-specific quantifications were generated, and raw estimated counts, as well as transcripts-per-million (TPM), and RPKM estimates were used in downstream analysis.

References

DeLuca, D. S., Levin, J. Z., Sivachenko, A., Fennell, T., Nazaire, M.-D., Williams, C., … Getz, G. (2012). RNA-SeQC: RNA-seq metrics for quality control and process optimization. *Bioinformatics (Oxford, England)*, *28*(11), 1530–1532.

Patro, R., Mount, S. M., & Kingsford, C. (2014). Sailfish enables alignment-free isoform quantification from RNA-seq reads using lightweight algorithms., *32*(5), 462–464.
