# Supplementary material for: Applications of Bayesian network models in predicting types of hematological malignancies
Source: Sci Rep. 2018 May 3;8:6951. doi: 10.1038/s41598-018-24758-5 (PMC5934387; doi:10.1038/s41598-018-24758-5)
Supplement: Supplementary file 10 — Supplementary Note S1 [file 41598_2018_24758_MOESM10_ESM.pdf]

Applications of Bayesian network models in predicting types of hematological malignancies

Rupesh Agrahari[1,+], Amir Foroushani[1,+], Thomas Roderick Docking[2], Linda Chang[2], Gerben Duns[2], Monika Hudoba[3], Aly Karsan[2,‡], and Habil Zare[2,‡,*]

[1]Department of Computer Science, Texas State University, San Marcos, Texas, 78666, USA
[2]Department of Pathology and Laboratory Medicine, British Columbia Cancer Agency, Vancouver, British Columbia, V5Z 4E6, Canada
[3]Department of Pathology and Laboratory Medicine, Vancouver General Hospital, Vancouver, British Columbia, V5Z 1M9, Canada
[*]zare@txstate.edu
[+]These authors contributed equally to this work.
[+]These senior authors contributed equally to this work.

Supplementary Note 1: Alternative approaches in Bayesian network analysis.

We implemented one of many ways in which a Bayesian network could be designed, trained, and used to infer information from eigengenes. We discuss some of the prominent alternative approaches below.

1. **The BN design:** In this study, the disease type is modeled by the *Effect* node, which is a binary random variable that cannot have any children by construction. An alternative design could allow this node to have children but no parents. Our preliminary results suggest that both of these designs lead to similar accuracy for the classification of AML vs. MDS (data not shown). However, we believe this could be due the relatively strong features we used (Fig 3); therefore, further experiments with other datasets are needed to determine the superior design.

   One argument in favor of the alternative design is that it can model the variables that are independent conditioned on the disease type. For simplicity, assume that (a) the model consists of only two eigengenes, each corresponding to a biological pathway that is inactive in MDS, and (b) these two biological pathways are independently active in AML. Then, the corresponding eigengenes are conditionally independent given the disease. While this is a plausible biological scenario, the first design cannot model this kind of probabilistic dependencies. In contrast, the alternative design can model a naïve Bayes classifier, that is, a network in which the two eigengenes are the children of the disease node and there is no edge between them. In this model, eigengenes are conditionally independent because they are d-separated.[1] The `learn.bn` function of the Pigengene package can implement the alternative design using `use.Disease=TRUE` and `use.Effect=FALSE`.

2. **Discretization:** Early attempts to use Bayesian networks for modeling gene expression data involved discretizing the level of expression to avoid computationally prohibitive calculations over continuous distributions.[2] Using current common computational resources, the *bnlearn* package can learn the structure of a BN in which each node is a continuous, Gaussian random variable. While this approach avoids the possible loss of information due to discretization, applying it in this study required that we assumed that the distribution of the eigengenes is Gaussian (normal). Furthermore, Friedman *et al.* reported that the two discrete and continuous methods highlight different types of connections between genes.[3] In applying our approach on other datasets that may be better modeled using continuous distributions, we recommend that a normality test is applied first.[4] If the distribution of the eigengenes fails the test, then one should use a proper transformation,[5,6] such as the log transformation, the Box-Cox transformation,[7] quantile normalization,[8] or rank normalization.[9,10]

3. **Inference:** To predict the value of the *Effect* node, we used the *bnlearn* package (Version 4.0), and we set `method=bayes-lw`. With this setting, *bnlearn* uses *likelihood weighting*,[11,12] which is an importance sampling algorithm.[13] That is, *bnlearn* averages 500 likelihood weighting simulations performed using all the available nodes as evidence. An alternative approach would be to export the BN model that was fitted by *bnlearn*, and use it as an input to other tools that are more suited for exact or approximate inference, such as JAGS,[14] OpenBUGS,[15] and Stan.[16] Some of these powerful tools have very recent R interfaces. Alternative approximate inference algorithms include stochastic Markov Chain Monte Carlo (MCMC),[17,18] mini-bucket elimination,[19] loopy belief propagation,[20] generalized belief propagation,[21] and variational methods.[22] In the specific BN design that we presented here, inference is relatively simple. That is, because the *Effect* node has no children by construction, conditioned on its parents, which are all observed random variables, *Effect* is independent from the rest of the network. Therefore, we expect that similar results would be obtained from our BN design if alternative inference algorithms were used.

References

1. Koller, D. & Friedman, N. *Probabilistic graphical models: principles and techniques* (MIT press, 2009).

2. Yu, J., Smith, V., Wang, P. P., Hartemink, A. J. & Jarvis, E. D. Using bayesian network inference algorithms to recover molecular genetic regulatory networks. In *International Conference on Systems Biology*, vol. 2002 (2002).

3. Friedman, N., Linial, M., Nachman, I. & Pe'er, D. Using Bayesian networks to analyze expression data. *J. Comput. Biol.* **7**, 601–620 (2000).

4. Ghasemi, A. & Zahediasl, S. Normality tests for statistical analysis: a guide for non-statisticians. *International journal of endocrinology and metabolism* **10**, 486 (2012).

5. Zwiener, I., Frisch, B. & Binder, H. Transforming rna-seq data to improve the performance of prognostic gene signatures. *PloS one* **9**, e85150 (2014).

6. Qiu, X., Wu, H. & Hu, R. The impact of quantile and rank normalization procedures on the testing power of gene differential expression analysis. *BMC bioinformatics* **14**, 124 (2013).

7. Box, G. E. & Cox, D. R. An analysis of transformations. *Journal of the Royal Statistical Society. Series B (Methodological)* 211–252 (1964).

8. Bolstad, B. M., Irizarry, R. A., Åstrand, M. & Speed, T. P. A comparison of normalization methods for high density oligonucleotide array data based on variance and bias. *Bioinformatics* **19**, 185–193 (2003).

9. Tsodikov, A., Szabo, A. & Jones, D. Adjustments and measures of differential expression for microarray data. *Bioinformatics* **18**, 251–260 (2002).

10. Szabo, A. *et al.* Variable selection and pattern recognition with gene expression data generated by the microarray technology. *Mathematical Biosciences* **176**, 71–98 (2002).

11. Fung, R. M. & Chang, K.-C. Weighing and integrating evidence for stochastic simulation in bayesian networks. In *Proceedings of the Fifth Annual Conference on Uncertainty in Artificial Intelligence*, 209–220 (North-Holland Publishing Co., 1990).

12. Shachter, R. D. & Peot, M. A. Simulation approaches to general probabilistic inference on belief networks. In *Proceedings of the Fifth Annual Conference on Uncertainty in Artificial Intelligence*, 221–234 (North-Holland Publishing Co., 1990).

13. Rubinstein, R. Y. & Kroese, D. P. *Simulation and the Monte Carlo method*, vol. 10 (John Wiley & Sons, 2016).

14. Denwood, M. J. runjags: An r package providing interface utilities, model templates, parallel computing methods and additional distributions for mcmc models in jags. *Journal of Statistical Software* **71**, 1–25 (2016).

15. Lunn, D., Spiegelhalter, D., Thomas, A. & Best, N. The bugs project: evolution, critique and future directions. *Statistics in medicine* **28**, 3049–3067 (2009).

16. Carpenter, B. *et al.* Stan: A probabilistic programming language. *Journal of Statistical Software* **76**, 1–32 (2017).

17. Pearl, J. Evidential reasoning using stochastic simulation of causal models. *Artificial Intelligence* **32**, 245–257 (1987).

18. Chavez, R. M. & Cooper, G. F. A randomized approximation algorithm for probabilistic inference on bayesian belief networks. *Networks* **20**, 661–685 (1990).

19. Dechter, R. & Rish, I. Mini-buckets: A general scheme for bounded inference. *Journal of the ACM (JACM)* **50**, 107–153 (2003).

20. Murphy, K. P., Weiss, Y. & Jordan, M. I. Loopy belief propagation for approximate inference: An empirical study. In *Proceedings of the Fifteenth conference on Uncertainty in artificial intelligence*, 467–475 (Morgan Kaufmann Publishers Inc., 1999).

21. Yedidia, J. S., Freeman, W. T. & Weiss, Y. Understanding belief propagation and its generalizations. *Exploring artificial intelligence in the new millennium* **8**, 236–239 (2003).

22. Jordan, M. I., Ghahramani, Z., Jaakkola, T. S. & Saul, L. K. Introduction to variational methods for graphical models. *Machine Learning* **37**, 183–233 (1999).
