# Supplementary material for: Applications of Bayesian network models in predicting types of hematological malignancies
Source: Sci Rep. 2018 May 3;8:6951. doi: 10.1038/s41598-018-24758-5 (PMC5934387; doi:10.1038/s41598-018-24758-5)
Supplement: Supplementary file 11 — Supplementary File descriptions [file 41598_2018_24758_MOESM11_ESM.pdf]

Applications of Bayesian network models in predicting types of hematological malignancies

Rupesh Agrahari[1,+], Amir Foroushani[1,+], Thomas Roderick Docking[2], Linda Chang[2], Gerben Duns[2], Monika Hudoba[3], Aly Karsan[2,‡], and Habil Zare[2,‡,*]

[1]Department of Computer Science, Texas State University, San Marcos, Texas, 78666, USA
[2]Department of Pathology and Laboratory Medicine, British Columbia Cancer Agency, Vancouver, British Columbia, V5Z 4E6, Canada
[3]Department of Pathology and Laboratory Medicine, Vancouver General Hospital, Vancouver, British Columbia, V5Z 1M9, Canada
*zare@txstate.edu
+These authors contributed equally to this work.
+These senior authors contributed equally to this work.

Description of supplementary material

Supplementary File S1. The eigengene values in MILE and BCCA data useful for reproducing the results.

Supplementary File S2. The BN structures.

Supplementary File S3. The predicted disease type for each of the BCCA and MILE samples.

Supplementary File S4. The confusion matrices and performance of individual models obtained from subsampling.

Supplementary File S5. The details of library preparation and RNA-Seq analysis in the BCCA dataset.

Supplementary File S6. The the eigengenes computed using the METABRIC breast cancer datasets. The values of each eigengene is reported on one column. The vital status of the cases is also included, where “d-d.s.” denotes “death due to the disease” and “a” denotes “alive”. The predictions are on the last column.

Supplementary Table S1. Sensitivity analysis. Each column includes the result of a *WGCNA* analysis on a fraction of 9,166 genes, which is indicated by the column names. The overlaps of the original modules (rows) with the resulting modules is normalized by the size of the original modules.

Supplementary Table S2. The list of genes that are associated with the breast cancer according to the BN model. This gene module is the only parent of the *Effect* node.

Supplementary Note S1. Alternative approaches in Bayesian network analysis.
